# ^18^F-labelled triazolyl-linked argininamides targeting the neuropeptide Y Y_1_R for PET imaging of mammary carcinoma

**DOI:** 10.1038/s41598-019-49399-0

**Published:** 2019-09-10

**Authors:** Simone Maschauer, Julian J. Ott, Günther Bernhardt, Torsten Kuwert, Max Keller, Olaf Prante

**Affiliations:** 1 Department of Nuclear Medicine, Molecular Imaging and Radiochemistry, Friedrich-Alexander University (FAU), Schwabachanlage 6, 91054 Erlangen, Germany; 2 Institute of Pharmacy, Faculty of Chemistry and Pharmacy, University of Regensburg, Universitätsstrasse 31, 93053 Regensburg, Germany

**Keywords:** Cancer imaging, Drug discovery and development, Pharmaceutics

## Abstract

Neuropeptide Y Y_1_ receptors (Y_1_R) have been found to be overexpressed in a number of different tumours, such as breast, ovarian or renal cell cancer. In mammary carcinoma the high Y_1_R density together with its high incidence of 85% in primary human breast cancers and 100% in breast cancer derived lymph node metastases attracted special attention. Therefore, the aim of this study was the development of radioligands for Y_1_R imaging by positron emission tomography (PET) with a special emphasis on imaging agents with reduced lipophilicity to provide a PET ligand with improved biodistribution in comparison with previously published tracers targeting the Y_1_R. Three new radioligands based on BIBP3226, bearing an ^18^F-fluoroethoxy linker (**12**), an ^18^F-PEG-linker (**13**) or an ^18^F-fluoroglycosyl moiety (**11**) were radiosynthesised in high radioactivity yields. The new radioligands displayed Y_1_R affinities of 2.8 nM (**12**), 29 nM (**13**) and 208 nM (**11**) and were characterised *in vitro* regarding binding to human breast cancer MCF-7-Y1 cells and slices of tumour xenografts. *In vivo*, small animal PET studies were conducted in nude mice bearing MCF-7-Y1 tumours. The binding to tumours, solid tumour slices and tumour cells correlated well with the Y_1_R affinities. Although **12** and **13** showed displaceable and specific binding to Y_1_R *in vitro* and *in vivo*, the radioligands still need to be optimised to achieve higher tumour-to-background ratios for Y_1_R imaging by PET. Yet the present study is another step towards an optimized PET radioligand for imaging of Y_1_R *in vivo*.

## Introduction

Neuropeptide Y (NPY), peptide YY (PYY) and pancreatic polypeptide (PP) are 36 amino acid peptides forming a family of biologically active peptides, the so-called neuropeptide Y or pancreatic polypeptide family^1^. Today, five mammalian NPY receptors (YRs) are known from molecular cloning, the Y_1_R, Y_2_R, Y_4_R, Y_5_R and y_6_ receptor^2–4^. The y_6_ receptor gene encodes a functionally active protein in mice, but is a pseudogene in humans and not present in the rat genome at all^3^. All five subtypes belong to the large superfamily of G-Protein Coupled Receptors (GPCRs) which are characterised as proteins located in the cell membrane consisting of seven transmembrane helices. The binding affinity of endogenous ligands is quite inhomogeneous: while NPY strongly binds to Y_1_R, Y_2_R and Y_5_R (EC_50_ values in the single-digit nanomolar range) its affinity to the Y_4_R subtype is lower by at least two orders of magnitude. In contrast, PP is the only endogenous ligand binding to Y_4_R at low nanomolar concentrations^5^. NPY is one of the most abundant neuropeptides in the central and peripheral nervous system. It is involved in the regulation of numerous physiological and pathophysiological processes, such as gastro-intestinal regulation, food intake and blood pressure.

Recently, Y_1_R and Y_2_R have been found to be overexpressed in a number of different tumours, such as ovarian cancer (Y_1_R and Y_2_R)^6^, neuroblastoma (Y_2_R only)^7^ or renal cell carcinomas (Y_1_R only)^8^. In mammary carcinoma, not only the high incidence of Y_1_Rs of 85% in malignant primary human breast tumours and 100% in breast cancer derived lymph node metastases attracted special attention, but also the high receptor density. *In-vitro* autoradiography studies on tumour slices with [^125^I]hPYY and non-radioactive, subtype-selective ligands for Y_1_R and Y_2_R revealed high specific binding (up to >12000 dpm/mg) due to extremely high density of the receptors in breast cancer^9^. Interestingly, the neoplastic transformation comes along with a switch in YR subtype expression: while healthy breast tissue only expresses the subtype Y_2_R, the Y_1_R subtype was found in tumour tissue only or at least predominantly^9^. Therefore, the Y_1_R is a promising target for tumour diagnosis and targeted tumour therapy, due to the high receptor expression and the predominance of the Y_1_R subtype in breast cancer tissue.

As subtype selective ligands are highly attractive for specific targeting of Y_1_R-positive tumours, many efforts have been made to design such subtype selective ligands. In 2001, the Beck-Sickinger group reported the first Y_1_R-preferring NPY analogues. [Phe^7^,Pro^34^]pNPY showed the highest selectivity for Y_1_R over Y_2_R and Y_5_R (>1:3000-fold)^10^. This compound was further developed towards a ^99m^Tc-labelled radioligand, [^99m^Tc]Tc(CO)_3_-N^α^His-Ac-[Phe^7^,Pro^34^]-NPY, which was also used in first human imaging studies^11^. Based on this work, we have reported the first ^18^F-labelled analogue of NPY, [Pra^4^([^18^F]FGlc),Phe^7^,Pro^34^]NPY, which was synthesised by ^18^F-fluoroglycosylation using the corresponding alkyne-functionalised peptide^12^.

Besides the peptidic NPY analogues, a number of non-peptide Y_1_R ligands have been reported, such as BIBP3226^13^, BIBO3304^14^, LY357897^15^ and Y1-973^16^. The latter was radiolabelled with fluorine-18, a positron emitting radionuclide with beneficial decay characteristics, i.e. a half-life of 110 min, a clean decay profile (97% positron emission, 3% electron capture) and a low positron energy (max. 0.635 MeV), resulting a low maximum positron range of 2.4 mm in water and therefore leading to high-resolution positron emission tomography (PET) images^17^. [^18^F]Y1-973 was studied as the first non-peptide Y_1_R antagonist to be successfully applied for *in vivo* imaging of Y_1_R in the central nervous system (CNS) of monkeys^16^. However, this compound is most likely not suitable for peripheral imaging of breast cancer due to its high lipophilicity. The (*R*)-argininamide BIBP3226 was described as the first highly potent and selective Y_1_R antagonist in 1994^13^. Recently, we reported prototypic ^18^F-labelled argininamide-type Y_1_R antagonists derived from BIBP3226 as candidate radioligands for PET (**1**–**4**, Table 1)^18^. The most favorable compound (**1**^18^, Table 1) was an amine-functionalised carbamoyl-derivative of BIBP3226 which was synthesised by ^18^F-fluoroacylation using 4-nitrophenyl-2-[^18^F]fluoropropionate ([^18^F]NPFP). This ligand is highly potent (K_i_ (Y_1_R) = 1.3 nM) and subtype selective (>1:3000 over Y_2_R and >1:10000 over Y_4_R and Y_5_R) and showed excellent *in vivo*-stability in mice. In nude mice bearing Y_1_R-positive MCF-7 tumour xenografts, the compound showed a rapid blood clearance, but extraordinarily high accumulation in the gall bladder (>200%ID/g at 30 min p.i.). With only 0.51%ID/g at 30 min p.i., the tumour uptake was low, but the radioligand showed good retention in the tumour (0.43%ID/g at 90 min p.i.). In comparison to the hydrophilic peptide radioligand agonist [Pra^4^([^18^F]FGlc),Phe^7^,Pro^34^]NPY^12^, the uptake of the compound in the kidneys was reduced by a factor of 10, being the major advantage of the hydrophobic small molecule antagonist PET radioligand.

**Table 1:** Structures and Y_1_R binding affinities of BIBP3226, alkyne precursors **2**^18^ and **7**, and the potential Y_1_R selective PET ligands **1**, **3**^18^ and **11**–**13**.


compound	structure		K_i_ [nM]	±SEM	n
**BIBP3226** ^13^	R^1^		1.3	±0.2^a^	2
**1** (equals number 23 in ref.^18^)	R^1^		1.3	±0.4^a^	2
**2** (equals number 39 in ref.^18^)	R^1^		1.3	±0.6^a^	2
**3** (equals number 41 in ref.^18^)	R^1^		2000	±180^a^	2
**7**	R^1^		0.94	±0.11^b^	3
**11**	R^2^		208	±24^b^	3
**12**	R^2^		2.8	±0.7^b^	5
**13**	R^2^		29.0	±3.6^b^	3

Dissociation constants (K_i_) were determined by displacement of ^a^[^3^H]UR-MK114^19^ or ^b^[^3^H]UR-MK299^22^ at Y_1_R-expressing SK-N-MC neuroblastoma cells. Mean values ± SEM are given from n independent experiments performed in duplicate or triplicate.

Based on these findings, the aim of this study was to reduce the lipophilicity of a candidate ligand in order to achieve a more suitable biodistribution with reduced biliary excretion and thus a better visibility of the tumour in PET imaging studies. Therefore, we synthesised three BIBP3226-derivatives, two with ^18^F-fluoroethoxy-linkers and one with an ^18^F-fluoroglucosyl moiety, and compared their properties *in vitro* on MCF-7-Y1 cells and *in vivo* using a nude mouse tumour xenograft model.

## Results and Discussion

### Chemistry and radiochemistry

**Figure 1:**
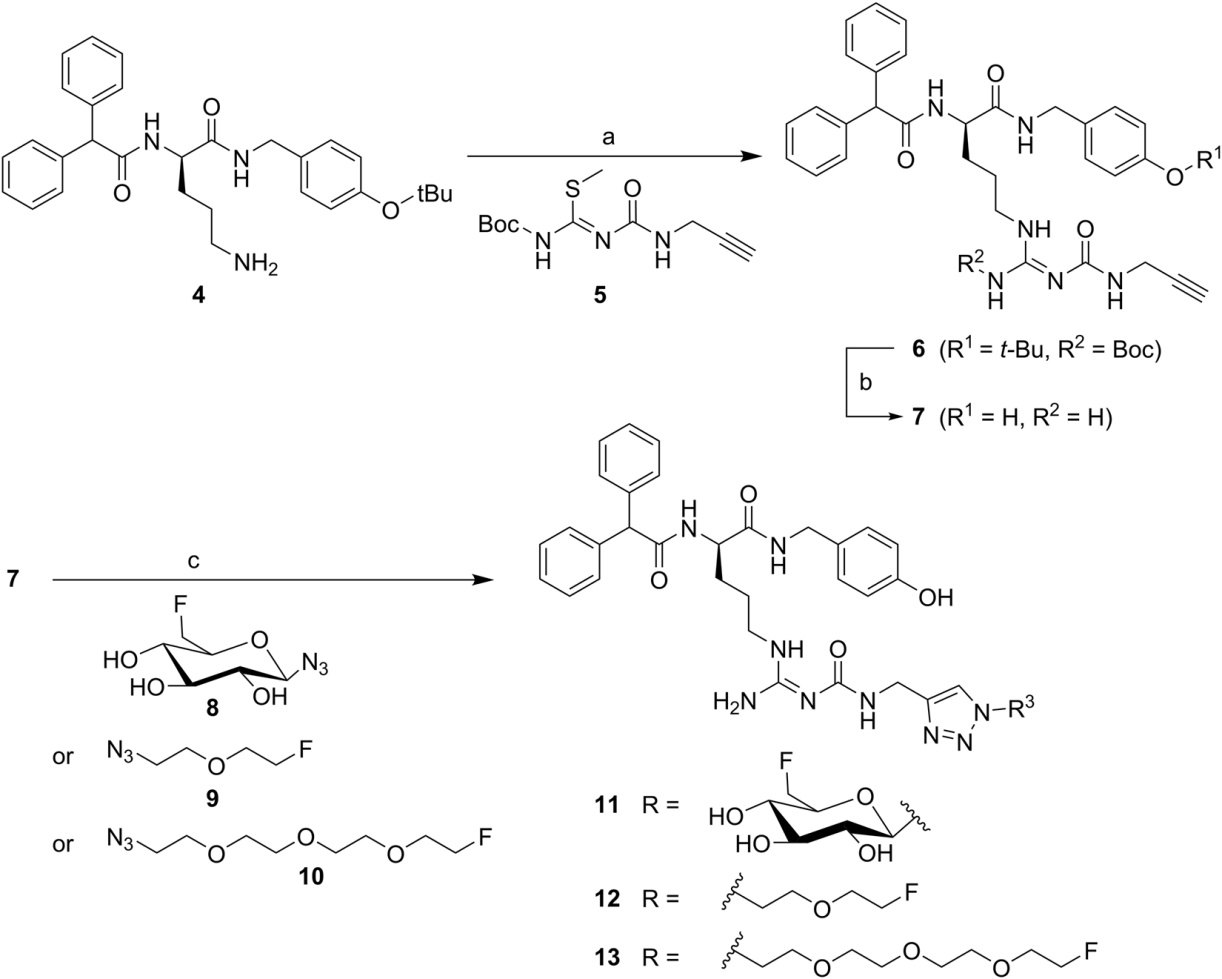
Synthesis of the alkyne-functionalised argininamide **7**, serving as a precursor for click chemistry-based ^18^F-labelling (see Fig. 2), and preparation of the potential Y_1_R ligands **11–13**. Reagents and chemical yields: (a) HgCl_2_, DMF, DIPEA, 26%; (b) TFA, CH_2_Cl_2_, quantitative; (c) copper(II) acetate, sodium ascorbate, solvent: *tert*-butanol/water (**11**) or *tert*-butanol/water/acetonitrile (**12**, **13**), 53% (**11**), 45% (**12**), 26% (**13**).

The alkynylated labelling precursor **7** was prepared from amine **4**^19^ by guanidinylation of **4** with the isothiourea derivative **5** in the presence of mercury(II) chloride yielding intermediate **6**, which was treated with trifluoroacetic acid (TFA) to obtain alkyne **7** (Fig. 1). The latter was subjected to copper(I)-catalysed cycloadditions with azides **8**^20^, **9** and **10** yielding the potential Y_1_R ligands **11**–**13** in a purity of >95% (Fig. 1).

**Figure 2:**
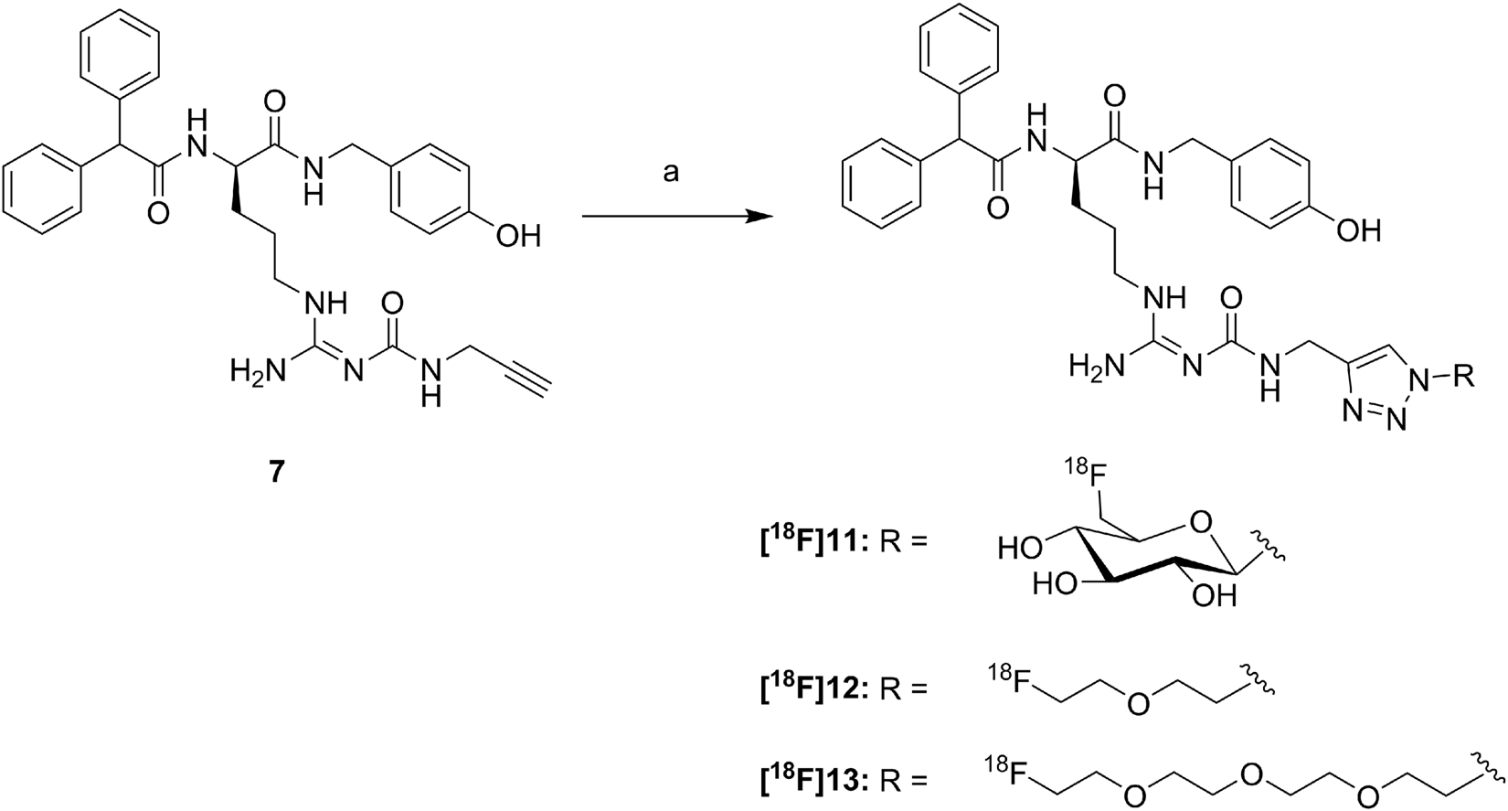
Radiosynthesis of **[**^**18**^**F]11, [**^**18**^**F]12** and **[**^**18**^**F]13**. Reagents and conditions: (a) **[**^**18**^**F]8**, **[**^**18**^**F]9** or **[**^**18**^**F]10**, Cu(OAc)_2_, sodium ascorbate, THPTA, pH 8, 60 °C, 15 min.

The radiosynthesis of **[**^**18**^**F]11** was performed according to a copper-catalysed azide-alkyne cycloaddition (CuAAC)-based ^18^F-fluoroglycosylation method (Fig. 2)^21^. The BIBP3226-derived alkyne **7** was treated with 6-deoxy-6-[^18^F]fluoroglucosyl azide **[**^**18**^**F]8** in an aqueous solution at 60 °C for 15 min providing **[**^**18**^**F]11** in a high radiochemical yield (RCY) of >80%. The product was isolated by semi-preparative radio-HPLC in a radioactivity yield (RAY) of about 20% (referred to [^18^F]fluoride) with a molar activity of 9 GBq/µmol at the end of synthesis (EOS). The radiochemical purity was analysed by radio-HPLC and was >99%.

The radiosyntheses of **[**^**18**^**F]12** and **[**^**18**^**F]13** followed the same protocol as for **[**^**18**^**F]11**, except that the deprotection step was omitted. Labelling of the tosylate-bearing precursors **20** or **21**, provided **[**^**18**^**F]9** and **[**^**18**^**F]10**, respectively, with excellent radiochemical yields in the range of 90% within 5 min. Radio-HPLC separation of the ^18^F-labelled fluoroethoxy azides **[**^**18**^**F]9** and **[**^**18**^**F]10** was achieved by radio-HPLC without any UV-peaks interfering with the radioactive product peak (see Supplementary Figs S1 and S2). The click chemistry reaction with BIBP3226-derived alkyne **7** was performed under similar reaction conditions as for the glucosyl derivative **[**^**18**^**F]11** leading to high RCY of >70%. After HPLC-isolation a RAY of about 5% for **[**^**18**^**F]12** and 10% for **[**^**18**^**F]13** (referred to [^18^F]fluoride) after 90 min, a molar activity of 5–6 GBq/µmol at EOS, and a radiochemical purity of >99% were achieved.

### *In-vitro* characterisation

For the determination of the Y_1_R affinities of **11**, **12** and **13** (synthesis see Fig. 1), competition binding assays were carried out using the radioligand [^3^H]UR-MK299 (K_d_ = 44 pM) on SK-N-MC neuroblastoma cells as described previously^22^. K_i_ values of **11–13** are provided in Table 1 together with reference values from literature for the previously published compounds **1**–**3** for comparison. The precursor alkyne **7** was found to bind to the Y_1_R with an affinity of 0.94 nM and thus exhibiting a similar receptor affinity as BIBP3226 and the fluoroacylated compound **1** previously published by Keller *et al*. (both K_i_ = 1.3 nM)^18^. The 6-deoxy-6-fluoroglycosyl derivative **11** bound with an affinity of 208 nM to the Y_1_ receptor, which is a 10-fold higher Y_1_R affinity compared to the 2-deoxy-2-fluoroglycosyl derivative **3** (K_i_ = 2000 nM, Table 1) which had a longer spacer moiety between the fluoroglycosyl moiety and the binding motif^18^. Although exhibiting a lower affinity to the Y_1_R by a factor of about 200 compared to the lead compound BIBP3226, this finding supported our hypothesis, that shortening the linker from ten to only three atoms (**3** vs. **11**, Table 1) would result in a less pronounced decrease in Y_1_R affinity. Compared to the glycosyl derivatives, the compounds bearing the less polar fluoroethoxy groups revealed higher Y_1_R affinities: The K_i_ value of **13** was determined to be 29 nM, which means that it has a sevenfold higher affinity than the fluoroglycosylated compound **11**, and the affinity of ligand **12** comprising the short fluoroethoxy chain increased even more by one order of magnitude (K_i_ = 2.8 nM).

To assess the YR subtype selectivity profiles of the three potential Y_1_R ligands **11**, **12** and **13**, the affinities to the other subtypes of the neuropeptide Y receptor, Y_2_R, Y_4_R and Y_5_R, were determined (Table S1, Supplementary Information). None of the three compounds showed considerable binding to one of the other subtypes within the experimental range of concentrations (maximum concentration of 10 µM), confirming the subtype selectivity for Y_1_R of the ligands under study (**11**–**13**).

The octanol-water distribution coefficients (logD_7.4_) of the radioligands were determined by the “shake flask” method and revealed a weak lipophilicity for all three compounds, being in good accordance to the calculated values (Table 2). As expected, the fluoroglycosylated compound **[**^**18**^**F]11** was the most hydrophilic one in this series (logD_7.4_ = 0.78, calculated: 0.43) followed by the fluoroethoxy compound with the longer chain (**[**^**18**^**F]13**, logD_7.4_ = 1.49, calculated: 1.94) and the compound with the shortest side chain, **[**^**18**^**F]12** (logD_7.4_ = 1.74, calculated: 2.03). Obviously, there was a strong influence of the bulkiness or hydrophilicity of the side chain on receptor affinity: The more hydrophilic or the more sterically demanding the side chain, the lower is the affinity to the Y_1_R. The results of the previously published ligands **1** (logD_7.4_ = 2.34; K_i_ = 1.3 nM) and **3** (clogD_7.4_ = 0.37; K_i_ = 20,000 nM)^18^ support this hypothesis.

**Table 2:** *In-vitro* characteristics of potential Y_1_R radioligands **[**^**18**^**F]11**, **[**^**18**^**F]12** and **[**^**18**^**F]13**.

Compound	clogD_7.4_ calculated^a^	logD_7.4_ experimental^b^	Free fraction in plasma^c^	Stability over 3 h in human serum^d^
**[** ^**18**^ **F]1** ^**e**^	3.4	2.34 ± 0.03	N/A	N/A
**[** ^**18**^ **F]11**	0.43	0.78 ± 0.005	42%	>99%
**[** ^**18**^ **F]12**	2.03	1.74 ± 0.070	38%	>99%
**[** ^**18**^ **F]13**	1.94	1.49 ± 0.003	37%	>99%

^a^ClogD_7.4_ values were calculated using the software Marvin Sketch (ChemAxon).

^b^Experimental logD_7.4_ values were determined by the “shake flask” method (n = 3, performed in triplicates).

^c^The binding to human plasma proteins was determined by means of gel filtration tubes (n = 3).

^d^The stability of the radiotracers in human serum was determined by radio-HPLC (n = 1).

^e^Values for **[**^**18**^**F]1** were taken from ref.^18^ for comparison.

N/A not available.

The binding of the radioligands to human plasma proteins was determined by gel filtration and revealed a high fraction of protein bound radioligand: less than half of the amount of radioactivity in plasma was unbound and thus freely available in the blood (42% of **[**^**18**^**F]11**, 38% of **[**^**18**^**F]12** and 37% of **[**^**18**^**F]13**, Table 2).

The stability of the radiotracers was evaluated *in vitro* by radio-HPLC: None of the three radioligands showed any radioactive degradation products within 3 h of incubation in human serum at 37 °C (see Supplementary Fig. S3 and Table 2).

**Figure 3:**
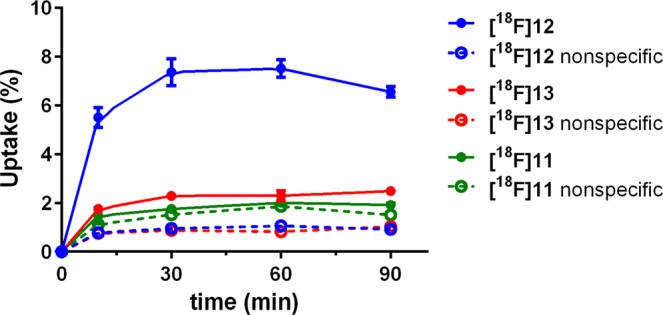
Total (solid line) and nonspecific (dashed line) uptake of the respective radioligands in MCF-7-Y1 cells. 300,000 cells/well were incubated with 15 kBq of the respective radioligand. Nonspecific uptake was determined by coincubation with 10 µM BIBP3226. Each point represents the mean ± standard deviation from one experiment performed in n = 6.

To determine cellular accumulation *in vitro*, assays with the ^18^F-labelled ligands were performed using the human breast cancer cells MCF-7-Y1. Cells were incubated either with the radioligand alone (total binding) or with the radioligand in the presence of 10 µM BIBP3226 as the blocking substance for determination of nonspecific binding. The total radioactivity in each well was defined as 100%. The highest specific accumulation was observed for the fluoroethoxy radioligand **[**^**18**^**F]12** (about 6% at 30 and 60 min, Fig. 3), whereas the fluoroethoxy radioligand **[**^**18**^**F]13** revealed a specific cellular association of only 1%, and the fluoroglycosylated radioligand **[**^**18**^**F]11** revealed almost no specific binding to MCF-7-Y1 cells (Fig. 3). This result can easily be explained by the lower affinities of **[**^**18**^**F]11** and **[**^**18**^**F]13** compared to the high-affinity ligand **[**^**18**^**F]12**.

**Figure 4:**
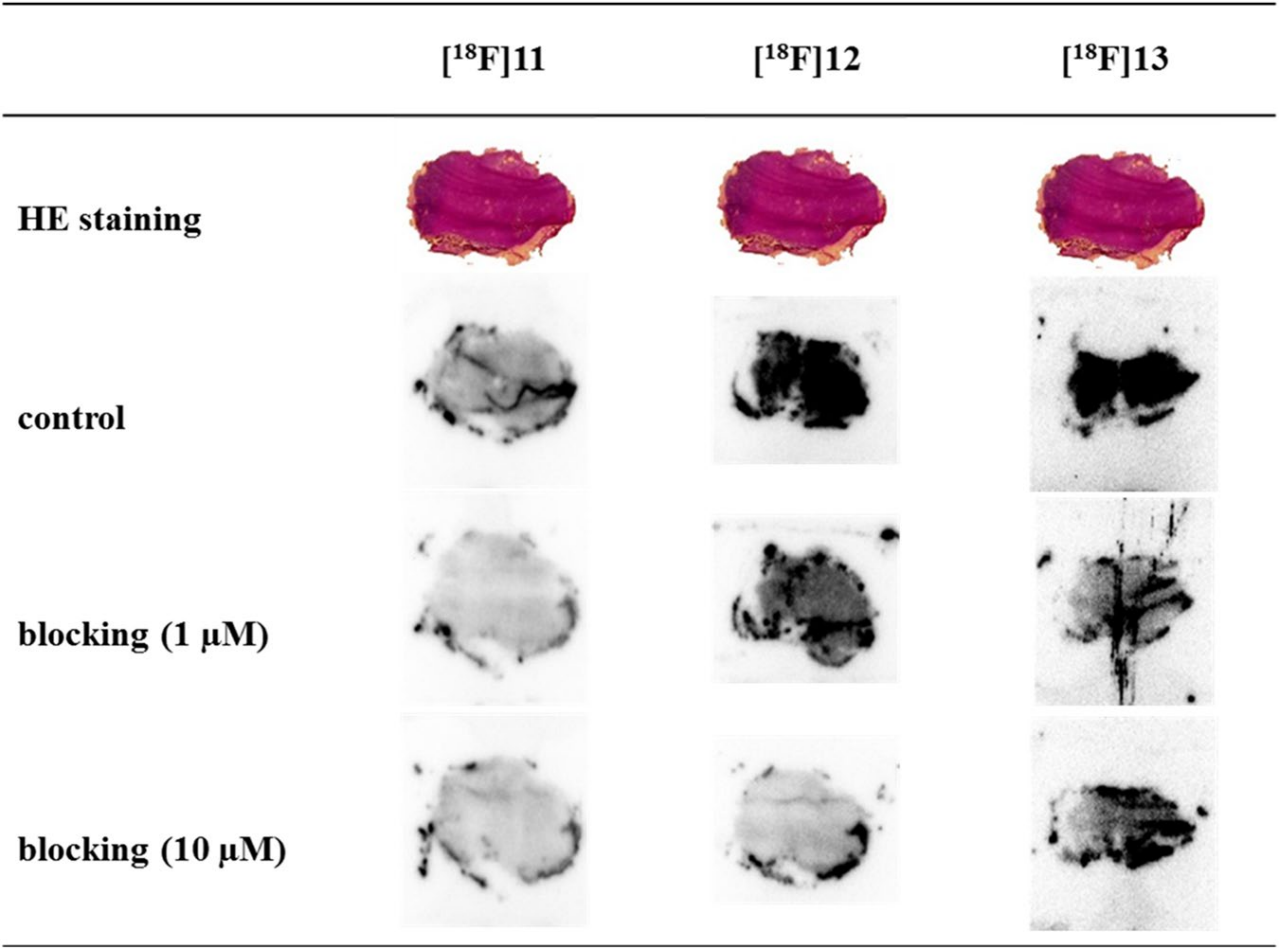
*In-vitro* autoradiography with the three potential Y_1_R radioligands **[**^**18**^**F]11**, **[**^**18**^**F]12** and **[**^**18**^**F]13** on MCF-7-Y1 tumour slices (14 µm) together with slices stained with HE. Blocking experiments were performed in the presence of 1 µM and 10 µM BIBP3226. Figure adapted from corresponding PhD thesis^26^.

To substantiate the specific binding of the radioligands to Y_1_R, autoradiography experiments were performed *in vitro* using slices of MCF-7-Y1 tumour xenografts. The tumour slices were incubated with each of the three radioligands in the presence or absence of BIBP3226 (1 µM and 10 µM). As expected, fluoroglycosylated ligand **[**^**18**^**F]11** showed very low specific binding to the tumour slices, whereas both fluoroethoxy ligands **[**^**18**^**F]12** and **[**^**18**^**F]13** showed marked specific binding to the solid MCF-7-Y1-tumours (Fig. 4).

### Biodistribution and small animal PET

**Figure 5:**
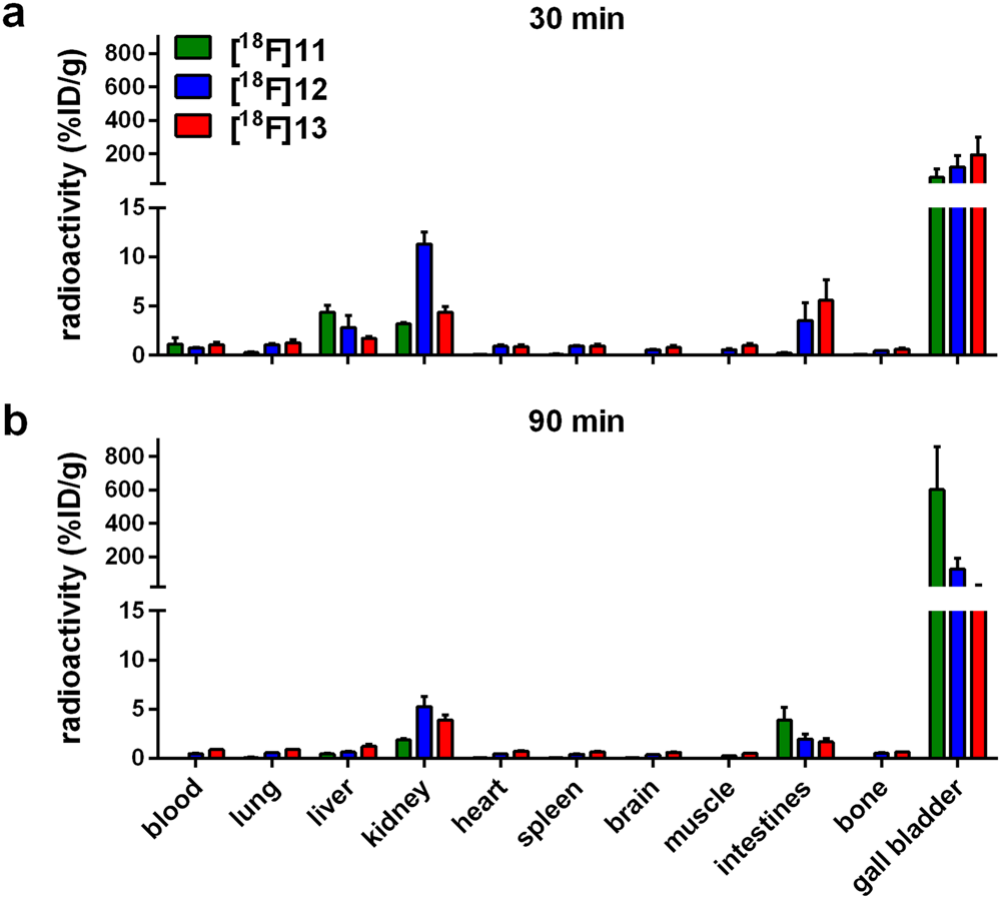
*In-vivo* biodistribution of the three Y_1_R radioligands **[**^**18**^**F]11**, **[**^**18**^**F]12** and **[**^**18**^**F]13** in normal mice a) 30 min and b) 90 min p.i. of the radiotracer. Data are given as mean ± SEM from three animals. Figure adapted from corresponding PhD thesis^26^.

The biodistribution of **[**^**18**^**F]11**, **[**^**18**^**F]12** and **[**^**18**^**F]13** was evaluated *in vivo* in healthy mice; the determined uptake values are depicted in Fig. 5. In general, the biodistribution of all three radioligands was very similar to that of **[**^**18**^**F]1**^18^. However, a detailed HPLC analysis of blood samples taken early after radiotracer injection revealed that the ^18^F-labelled radioligands **[**^**18**^**F]12** and **[**^**18**^**F]13** underwent rapid degradation in the blood, forming very hydrophilic radiometabolites, whereas the ^18^F-glycosyl derivative **[**^**18**^**F]11** interestingly revealed higher stability in the blood *in vivo* (Supplemantary Fig. S4). After 5 min p.i. only 10% of intact **[**^**18**^**F]12**, less than 5% of intact **[**^**18**^**F]13** and about 50% intact **[**^**18**^**F]11** were determined in the blood samples. The radioligands and radiometabolites showed fast clearance from the blood, as there was no detectable radioactivity in the blood at 90 min p.i. Moderate amounts of radioactivity were detected in the kidneys and intestines, and exceptionally high radioactivity was observed in the gall bladder (up to 600%ID/g after 90 min for **[**^**18**^**F]11**). All other organs did not show any significant accumulation of the radioligands. The uptake in the liver as the main organ for metabolism of xenobiotics was below 5%ID/g at 30 min and below 1.5%ID/g at 90 min p.i. The three ^18^F-radioligands revealed a more differentiated result regarding the uptake in the kidney, which can be ascribed to the formation of hydrophilic radiometabolites in the blood (Supplemantary Fig. S4): Whereas **[**^**18**^**F]11** and **[**^**18**^**F]13** revealed kidney uptake of <5%ID/g at 30 and 90 min p.i., the uptake of **[**^**18**^**F]12** was about three times higher. As reported earlier for **[**^**18**^**F]1**^18^, the majority of the injected radioactivity was found in the gall bladder (>100%ID/g) and in the intestines. For the glycosylated ligand **[**^**18**^**F]11** the excretion seems to occur more slowly as the accumulation in these organs increased from 30 to 90 min p.i., while it stayed constant or decreased in case of the fluoroethoxy ligands **[**^**18**^**F]12** and **[**^**18**^**F]13**. The high accumulation in the bile might hamper tumour imaging by PET, as it was previously described for **[**^**18**^**F]1**^18^. The aim of reducing the uptake in the gall bladder by using more hydrophilic radioligand analogues, was obviously not reached, although the lipophilicity of the radiotracers was in fact reduced. Since only very little radioactivity was detected in the bones, it is most likely that there was no cleavage of fluoride from the molecules in terms of metabolism.

**Figure 6:**
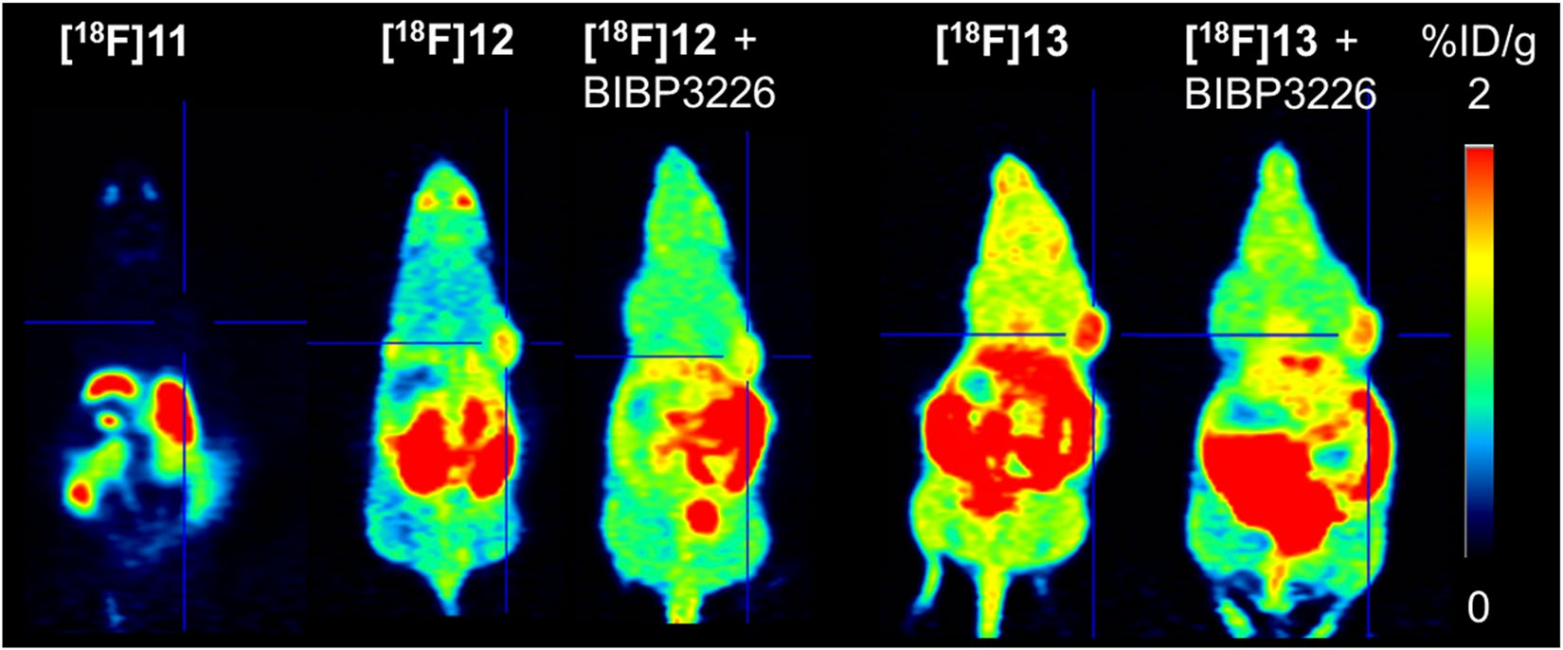
PET images of in MCF-7-Y1-tumour bearing nude mice injected with Y_1_R radioligands **[**^**18**^**F]11**, **[**^**18**^**F]12** and **[**^**18**^**F]13** at 45–60 min p.i. Tumours are indicated by crosshair. The blocking experiments were performed by co-injection of 1 mg/kg BIBP3226.

Finally, PET imaging was performed with the three radiotracers on MCF-7-Y1 xenografted nude mice. However, despite low background, visualision of the tumour with the fluoroglycosylated radiotracer **[**^**18**^**F]11** failed, most likely due to the low affinity of the radioligand to the Y_1_R (Fig. 6, left panel). Both fluoroethoxy radioligands **[**^**18**^**F]12** (Fig. 6, middle panel) and **[**^**18**^**F]13** (Fig. 6, right panel) demonstrated displaceable and thus specific Y_1_R-mediated tumour acculuation *in vivo*, which was proven by co-injection of the radioactive compounds together with the non-radioactive competitor BIBP3226 (1 mg/kg; Fig. 6, middle and right panel). The specific uptake of **[**^**18**^**F]13** in MCF-7 was less pronounced compared to **[**^**18**^**F]12**, nicely reflecting the *in-vitro* results on receptor binding data and cellular accumulation studies. Both radiotracers, **[**^**18**^**F]12** and **[**^**18**^**F]13**, showed relatively high background values in the PET images, compromising their further application *in vivo*. This may be explained by the fast formation of radiometabolites (>90%) in the blood (see Supplementary Information Fig. S4).

Compared to the previously published ligand **[**^**18**^**F]1**^18^, the radioligands **[**^**18**^**F]12** and **[**^**18**^**F]13** were more hydrophilic and demonstrated enhanced and specific tumour uptake leading to improved tumour-to-background-ratios, such that Y_1_R-positive tumours could clearly be visualised by PET. Nevertheless, there is still room for improvement for the design of an optimal radioligand for Y_1_R imaging by PET.

## Conclusion

Three BIBP3226-derivatives, two with ^18^F-fluoroethoxy-linkers and one with a ^18^F-fluoroglucosyl moiety, were radiosynthesised in sufficient radioactivity yields and molar activities. Dependent on the size of the carbamoyl residues attached to the guanidine group the three radioligands showed receptor affinities for Y_1_R from 2.8–208 nM. The radioligand with the highest affinity (**[**^**18**^**F]12**) revealed the highest specific binding to Y_1_R-positive cells and to tumour slices *in vitro*. Despite their different hydrophilicity (logD_7.4_ values ranging from 0.43 to 2.03), the biodistribution of the three radioligands in healthy mice was very similar. In PET scans of tumour-bearing mice, **[**^**18**^**F]12** showed the highest specific binding to the Y_1_R-positive tumour *in vivo*, corresponding to the highest *in-vitro* affinity of the ^18^F-labelled ligands under study. However, the PET imaging results suffered from high background levels, because of fast degradation of the radioligands in the blood and marked binding to plasma proteins. Therefore, the present study has to be regarded as another step towards the development of an optimal PET radioligand for Y_1_R imaging *in vivo*.

## Methods

### General

Radio-HPLC was performed on an Agilent 1100 system with a quarternary pump and a variable wavelength detector and a radio-HPLC detector D505TR (Canberra Packard). Computer analysis of the HPLC data was performed using FLO-One software (Canberra Packard). Electron-spray ionisation (ESI) mass spectrometry analysis was performed using a Bruker Esquire 2000 instrument.

### Chemistry

Synthetic procedures and analytical data for the guanidinylating reagent **5** and compounds **14**–**21** are described in the Supplementary Information associated with this article. The (R)-ornithine derivative **4**^19^ and the glycosyl azide **8**^20^ were prepared according to previously described procedures.

### (2*R*)‐5‐{[(1*Z*)‐Amino({[({1‐[(2*R*,5*S*)‐6‐(fluoromethyl)‐3,4,5‐trihydroxyoxan‐2‐yl]‐1H‐1,2,3‐triazol‐4‐yl}methyl)carbamoyl]imino})methyl]amino}‐2‐(2,2‐diphenylacetamido)‐*N*‐[(4‐hydroxyphenyl)-methyl]pentanamide (11)

**7** (5.54 mg, 10 µmol, 1 eq.) was dissolved in a 3-mL micro reaction vessel in 380 µL *tert*-butanol. 6-Deoxy-6-fluoro-β-D-glucopyranosyl azide **8** (6.21 mg, 30 µmol, 3 eq.) was dissolved in 232 µL water and added to the reaction vessel. Solutions of copper(II) acetate (67.5 µL, 40 mM) and sodium ascorbate (81.0 µL, 100 mM) were mixed for 1 min and then added to the reaction vessel. The solution was stirred for 30 min in the capped vessel. The mixture was then diluted with 20 mL water and passed through a preconditioned (10 mL acetonitrile, 15 mL water) Sep-Pak® C18 Plus Long SPE cartridge. The trapped product was eluted from the SPE cartridge with 2 mL ethanol and the solvent was evaporated *in vacuo*. The crude product was purified by semipreparative HPLC (Kromasil 100 C8 5 µm, 125 × 8 mm, 4 mL/min, 20–40% CH_3_CN (0.1% TFA) in water (0.1% TFA) in a linear gradient over 30 min, t_R_ = 16.23 min). The fractions containing the product were pooled, concentrated *in vacuo*, coevaporated with acetonitrile several times and dried *in vacuo*.

Yield: 4.0 mg as a brown oil (53%). HPLC: t_R_ = 3.46 min (Chromolith RP-18e, 100 × 4.6 mm, 4 mL/min, 10–90% CH_3_CN (0.1% TFA) in water (0.1% TFA) in a linear gradient over 10 min), 97%; t_R_ = 2.26 min (Chromolith RP-18e, 100 × 4.6 mm, 4 mL/min, 25% CH_3_CN (0.1% TFA) for 1 min, then 25–60% CH_3_CN (0.1% TFA) in water (0.1% TFA) in a linear gradient over 5 min), >98%; LC-MS: *m/z* calcd: 762.33 [M + H]^+^, found: 762.27 [M + H]^+^.

### (2*R*)‐5‐{[(1*Z*)‐Amino({[({1‐[2‐(2‐fluoroethoxy)ethyl]‐1H‐1,2,3‐triazol‐4‐yl}methyl)carbamoyl]-imino})methyl]amino}‐2‐(2,2‐diphenylacetamido)‐*N*‐[(4‐hydroxyphenyl)methyl]pentanamide (12)

**7** (5.54 mg, 10 µmol, 1 eq.) and **9** (6.00 mg, 45 µmol, 4.5 eq.) were dissolved in 380 µL *tert*-butanol and 232 µL water and added to a 3 mL micro reaction vessel. Solutions of copper(II) acetate (67.5 µL, 40 mM), sodium ascorbate (81.0 µL, 500 mM), THPTA (tris(3-hydroxypropyltriazolylmethyl)amine, 40 µL, 20 mM, in EtOH) and DIPEA (2 µL) were mixed for 1 min and then added to the reaction vessel. Acetonitrile (250 µL) was added to the reaction vessel and the solution was stirred for 5 min in the capped vessel. The mixture was diluted with 20 mL water and passed through a preconditioned (10 mL acetonitrile, 15 mL water) Sep-Pak® C18 Plus Long SPE cartridge. The trapped product was eluted from the SPE cartridge with 2 mL ethanol and the solvent was evaporated *in vacuo*. The crude product was purified by semipreparative HPLC (Kromasil 100 C8 5 µm, 125 × 8 mm, 4 mL/min, 30–40% CH_3_CN (0.1% TFA) in water (0.1% TFA) in a linear gradient over 30 min, t_R_ = 10.7 min). The fractions containing the product were pooled, concentrated *in vacuo*, coevaporated with acetonitrile several times and dried *in vacuo*.

Yield: 1.8 mg (26%); HPLC: t_R_ = 3.85 min (Chromolith RP-18e, 100 × 4.6 mm, 4 mL/min, 10–90% CH_3_CN (0.1% TFA) in water (0.1% TFA) in a linear gradient over 10 min), 95%; t_R_ = 2.96 min (Chromolith RP-18e, 100 × 4.6 mm, 4 mL/min, 25% CH_3_CN (0.1% TFA) for 1 min, then 25–60% CH_3_CN (0.1% TFA) in water (0.1% TFA) in a linear gradient over 5 min), 93%; LC-MS: *m/z* calcd: 688.33 [M + H]^+^, found: 688.24 [M + H]^+^.

### (2*R*)‐5‐{[(1*Z*)‐Amino[({[1‐(2‐{2‐[2‐(2‐fluoroethoxy)ethoxy]ethoxy}ethyl)‐1H‐1,2,3‐triazol‐4‐yl]methyl}carbamoyl)imino]methyl]amino}‐2‐(2,2‐diphenylacetamido)‐*N*‐[(4‐hydroxyphenyl)-methyl]pentanamide (13)

**7** (5.54 mg, 10 µmol, 1 eq.) and **10** (6.63 mg, 30 µmol, 3 eq.) were dissolved in 380 µL *tert*-butanol and 232 µL water and added to a 3 mL micro reaction vessel. Solutions of copper(II) acetate (67.5 µL, 40 mM), sodium ascorbate (81.0 µL, 500 mM), THPTA (40 µL, 20 mM, in EtOH) and DIPEA (2 µL) were mixed for 1 min and then added to the reaction vessel. Acetonitrile (250 µL) was added to the reaction vessel and the solution was stirred for 5 min in the capped vessel. The mixture was diluted with 20 mL water and passed through a preconditioned (10 mL acetonitrile, 15 mL water) Sep-Pak® C18 Plus Long SPE cartridge. The trapped product was eluted from the SPE cartridge with 2 mL ethanol and the solvent was evaporated *in vacuo*. The crude product was purified by semipreparative HPLC (Kromasil 100 C8 5 µm, 125 × 8 mm, 4 mL/min, 30–40% CH_3_CN (0.1% TFA) in water (0.1% TFA) in a linear gradient over 30 min, t_R_ = 11.5 min). The fractions containing the product were pooled, concentrated *in vacuo*, coevaporated with acetonitrile several times and dried *in vacuo*.

Yield: 3.5 mg (45%); HPLC: t_R_ = 3.93 min (Chromolith RP-18e, 100 × 4.6 mm, 4 mL/min, 10–90% CH_3_CN (0.1% TFA) in water (0.1% TFA) in a linear gradient over 10 min), 98%; t_R_ = 3.06 min (Chromolith RP-18e, 100 × 4.6 mm, 4 mL/min, 25% CH_3_CN (0.1% TFA) for 1 min, then 25–60% CH_3_CN (0.1% TFA) in water (0.1% TFA) in a linear gradient over 5 min), 97%; LC-MS: *m/z* calcd: 776.39 [M + H]^+^, found: 776.32 [M + H]^+^.

#### *In-vitro* determination of receptor affinity

Radioligand competition binding studies at the hY_1_R were performed on SK-N-MC neuroblastoma cells as previously described using the radioligand [^3^H]UR-MK299 (K_d_ (Y_1_R) = 0.044 nM)^22^. Competition binding experiments at the hY_2_R and hY_5_R were performed on CHO-hY_2_-G_qi5_-mtAEQ cells and HEC-1b hY_5_R cells, respectively, as previously reported using [^3^H]propionyl-pNPY (K_d_ (Y_2_R) = 1.4 nM, K_d_ (Y_5_R) = 4.8 nM) as radioligand^23^. Competition binding studies at the hY_4_R were performed on CHO-hY_4_R-G_qi5_-mtAEQ cells as previously described using [^3^H]UR-KK200 (K_d_ = 0.67 nM) as radioligand^23^. Data analysis (four-parameter sigmoidal fitting of specifically bound radioligand plotted against log(concentration) competitor) was performed using SigmaPlot (Systat Software, San Jose, CA, USA) (Y_1_R binding). IC_50_ values were converted to K_i_ values according to the Cheng-Prusoff equation^24^.

## Radiochemistry

### 1‐Azido‐2‐(2‐[^18^F]fluoroethoxy)ethane ([^18^F]9)

[^18^F]Fluoride was eluted from an anion-exchange cartridge (QMA, Waters) with a solution of 10 mg Kryptofix® 2.2.2., 18 µL 0.1 M K_2_CO_3_ and 18 µL 0.1 M KH_2_PO_4_ in 1 mL acetonitrile/water (8:2 v/v). The solvent was evaporated in a stream of nitrogen at 85 °C and co-evaporated to dryness with acetonitrile (3 × 0.5 mL). The labelling precursor **20** (9 mg, 32 µmol, kept under vacuum at 40 °C overnight) in anhydrous acetonitrile (0.45 mL) was added, and the mixture was stirred at 85 °C for 5 min. The crude reaction mixture was diluted with 0.5 mL of acetonitrile/water (1:4 v/v, 0.1% TFA) and submitted to semipreparative HPLC (Kromasil 100 C8 5 µm, 125 × 8 mm, 4 mL/min, 10–50% acetonitrile (0.1% TFA) in water (0.1% TFA) in a linear gradient over 25 min, t_R_ (**20**) = 14.8 min, t_R_ (**[**^**18**^**F]9**) = 8.1 min). The product fraction was diluted with water to a total volume of 20 mL and passed through a Strata-X® (Phenomenex, 100 mg/3 mL) SPE column. The product **[**^**18**^**F]9** was eluted with 2 mL THF and fractions of about 0.1 mL each were collected. Starting from 1370 MBq [^18^F]fluoride, this procedure yielded 527 MBq (38% radioactivity yield (RAY)) **[**^**18**^**F]9** in a total synthesis time of 40 min.

### 1‐Azido‐2‐{2‐[2‐(2‐[^18^F]fluoroethoxy)ethoxy]ethoxy}ethane ([^18^F]10)

[^18^F]Fluoride was eluted from an anion-exchange cartridge (QMA, Waters) with a solution of 10 mg Kryptofix® 2.2.2., 18 µL 0.1 M K_2_CO_3_ and 18 µL 0.1 M KH_2_PO_4_ in 1 mL acetonitrile/water (8:2 v/v). The solvent was evaporated in a stream of nitrogen at 85 °C and co-evaporated to dryness with acetonitrile (3 × 0.5 mL). The labelling precursor **21** (9 mg, 24 µmol, kept under vacuum at 40 °C overnight) in anhydrous acetonitrile (450 µL) was added and the mixture was stirred for 5 min at 85 °C. The solvent was again evaporated in a stream of nitrogen, and the residue was re-dissolved in 0.5 mL of acetonitrile/water (1:1 v/v, 0.1% TFA). The solution was submitted to semipreparative HPLC (Kromasil 100 C8 5 µm, 125 × 8 mm, 4 mL/min, 10–50% acetonitrile (0.1% TFA) in water (0.1% TFA) in a linear gradient over 25 min, t_R_ (**21**) = 14.2 min, t_R_ (**[**^**18**^**F]10**) = 10.3 min). The product fraction was diluted with water to a total volume of 20 mL and passed through a Strata-X® (Phenomenex, 100 mg/3 mL) SPE column. The product **[**^**18**^**F]10** was eluted with 1 mL ethanol. Starting from 918 MBq [^18^F]fluoride, this procedure yielded 369 MBq (40% RAY) **[**^**18**^**F]10** in a total synthesis time of 35 min.

### (2R)‐5‐{[(1Z)‐Amino({[({1‐[(2R,5S)‐6‐([^18^F]fluoromethyl)‐3,4,5‐trihydroxyoxan‐2‐yl]‐1H‐1,2,3‐triazol‐4‐yl}methyl)carbamoyl]imino})methyl]amino}‐2‐(2,2‐diphenylacetamido)‐N‐[(4‐hydroxyphenyl)-methyl]pentanamide ([^18^F]11)

2,3,4-Tri-*O*-acetyl-6-deoxy-6-[^18^F]fluoroglucosyl azide was prepared, isolated by semi-preparative HPLC and deacetylated with NaOH (250 µL, 60 mM, 10% ethanol) at 60 °C for 5 min as described before^20^. The crude product 6-deoxy-6-[^18^F]fluoroglucosyl azide **[**^**18**^**F]8** was subsequently used for the click chemistry reaction with alkyne **7** in a one-pot-procedure as described before^21^. In brief, to the solution containing **[**^**18**^**F]8** (in 270 µL of 60 mM NaOH and 30 µL ethanol) was given a mixture of 30 µL 20 mM THPTA, 30 µL 4 mM Cu(OAc)_2_, 30 µL 0.1 M sodium ascorbate, 25 µL **7** (200 nmol in ethanol), 270 µL 0.5 M phosphate buffer pH 8 and 50 µL ethanol. The mixture was stirred at 60 °C for 20 min. Subsequently, 350 µL of acetonitrile/water (1:1 v/v, 0.1% TFA) were added, and the solution was submitted to semipreparative HPLC (Kromasil 100 C8 5 µm, 125 × 8 mm, 4 mL/min, 20–40% acetonitrile (0.1% TFA) in water (0.1% TFA) in a linear gradient over 30 min, t_R_ (**7**) = 23.1 min, t_R_ (**[**^**18**^**F]11**) = 16.7 min). The product fraction was diluted with water to a total volume of 20 mL and passed through a Sep-Pak® C18 Plus Light SPE cartridge (Waters). The cartridge was washed with 5 mL water and **[**^**18**^**F]11** was eluted with 1 mL of ethanol/saline (1:1 v/v). The volume was reduced *in vacuo*. Starting from 430 MBq 2,3,4-tri-*O*-acetyl-6-deoxy-6-[^18^F]fluoroglucosyl azide, this procedure yielded 200 MBq (47% RAY) **[**^**18**^**F]11** in a total synthesis time of 80 min with a molar radioactivity of 9 GBq/µmol.

### (2R)‐5‐{[(1Z)‐Amino({[({1‐[2‐(2‐[^18^F]fluoroethoxy)ethyl]‐1H‐1,2,3‐triazol‐4‐yl}methyl)carbamoyl]-imino})methyl]amino}‐2‐(2,2‐diphenylacetamido)‐N‐[(4‐hydroxyphenyl)methyl]pentanamide ([^18^F]12)

30 µL 20 mM THPTA, 30 µL 4 mM Cu(OAc)_2_, and 30 µL 0.1 M sodium ascorbate were mixed in an Eppendorf reaction vessel for 1 min, followed by addition of 25 µL **7** (200 nmol in ethanol) and 270 µL 0.5 M phosphate buffer pH 8. The solution was mixed and added to a solution of **[**^**18**^**F]9** in ca. 0.3 mL of THF at 60 °C. The mixture was stirred at 60 °C for 20 min prior to dilution with 0.4 mL of water (containing 0.1% TFA). The mixture was submitted to semipreparative HPLC (Kromasil 100 C8 5 µm, 125 × 8 mm, 4 mL/min, 20–40% acetonitrile (0.1% TFA) in water (0.1% TFA) in a linear gradient over 30 min, t_R_ (**[**^**18**^**F]9**) = 6.3 min, t_R_ (**7**) = 23.1 min, t_R_ (**[**^**18**^**F]12**) = 22.0 min), the product fraction was diluted with water to a total volume of 20 mL and passed through a Sep-Pak® C18 Plus Light SPE cartridge (Waters). The cartridge was washed with 5 mL water and **[**^**18**^**F]12** was eluted with 1 mL ethanol/saline (1:1 v/v). The volume was reduced *in vacuo*. Starting from 104 MBq **[**^**18**^**F]9**, this procedure yielded 17 MBq (16% RAY) **[**^**18**^**F]12** in a total synthesis time of 60 min.

### (2R)‐5‐{[(1Z)‐Amino[({[1‐(2‐{2‐[2‐(2‐[^18^F]fluoroethoxy)ethoxy]ethoxy}ethyl)‐1H‐1,2,3‐triazol‐4‐yl]methyl}carbamoyl)imino]methyl]amino}‐2‐(2,2‐diphenylacetamido)‐N‐[(4‐hydroxyphenyl)-methyl]pentanamide ([^18^F]13)

A solution of **[**^**18**^**F]10** in ethanol was evaporated in a stream of nitrogen at 60 °C. The solution containing the components for the CuAAC reaction was prepared as follows: 30 µL 20 mM THPTA, 30 µL 4 mM Cu(OAc)_2_, and 30 µL 0.1 M sodium ascorbate were mixed in an Eppendorf tube for 1 min, followed by addition of 25 µL **7** (200 nmol in ethanol) and 270 µL 0.5 M phosphate buffer pH 8. The CuAAC solution was mixed and added to the dried residue. The mixture was stirred at 60 °C for 15 min. 0.2 mL acetonitrile/water (1:1 v/v, 0.1% TFA) were added and the solution was submitted to semipreparative HPLC (Kromasil 100 C8 5 µm, 125 × 8 mm, 4 mL/min, 20–40% acetonitrile (0.1% TFA) in water (0.1% TFA) in a linear gradient over 30 min, t_R_ (**[**^**18**^**F]10**) = 7.4 min, t_R_ (**7**) = 23.1 min, t_R_ (**[**^**18**^**F]13**) = 22.8 min). The product fraction was diluted with water to a total volume of ca. 20 mL and passed through a Sep-Pak® C18 Plus Light SPE cartridge. The cartridge was washed with 5 mL water and **[**^**18**^**F]13** was eluted with 1 mL ethanol/saline (1:1 v/v). The volume was reduced *in vacuo*. Starting from 369 MBq **[**^**18**^**F]10**, this procedure yielded 91 MBq (25% RAY) **[**^**18**^**F]13** in a total synthesis time of 60 min with a molar radioactivity of 5 GBq/µmol.

### *In-vitro* characterisation of radiotracers

#### Lipophilicity determination

The lipophilicity of the radioligands was determined by the distribution coefficient *logD*_*7.4*_. The respective radioligand (10 µL, about 25 kBq) was added to a mixture of PBS (500 µL) and 1-octanol (500 µL) and the emulsion was vortexed for 1 min. After centrifugation 3 × 100 µL were taken of each layer and analysed by a γ-counter. The partition coefficient was calculated as $log{D}_{7.4}=\,\log (\frac{cp{m}_{octanol}}{cp{m}_{PBS}})$. Data were expressed as mean values ± SD from three experiments.

#### Determination of plasma protein binding

The binding of ^18^F-labelled compounds to plasma proteins was determined using gel filtration columns(illustra^™^ MicroSpin^™^ G-50 Columns, GE Healthcare Life Sciences, Freiburg). An aliquot of the radiotracer (approx. 100 kBq) was added to 200 µL of saline, and 100 µL of human plasma, respectively. Both samples were incubated at 37 °C for 10 min. MicroSpin^™^ columns were prepared according to the user instruction. Then 25 µL of the incubated radiotracer were given onto the columns and the devices were centrifuged (2000 × g, 2 min). Eluate and solid phase were analysed in the γ-counter (Wallac Wizard). The percentage of unbound radioligand was calculated as $\% \,free=\frac{cp{m}_{solidphase}}{cp{m}_{solidphase}+cp{m}_{eluate}}$. The sample in saline was used as control.

#### Determination of radiotracer stability in human serum

The stability of ^18^F-labelled compounds was determined in human serum. An aliquot of the radiotracer (5–10 MBq) was added to 200 µL human serum and incubated at 37 °C. Aliquots of 15 µL were taken after 5, 10, 15, 30, 45, 60, 120 and 180 min and quenched in 100 µL of 10% aqueous TFA. The samples were centrifuged (20000 × g, 2 min), and the supernatants were analysed by radio-HPLC (Chromolith RP-18e, 100 × 4.6 mm, 4 mL/min, 0–1 min 25% CH_3_CN (0.1% TFA) in water (0.1% TFA), 1–6 min 25–60%, 6–7 min 60–100%, 7–8 min 100%).

#### Cell line

MCF-7-Y1 is a subclone that originated in Prof. Dr. Armin Buschauer’s group (Regensburg University, Regensburg) from human breast cancer cell line MCF-7 in the 157^th^ passage and shows 2–3 fold higher Y_1_R expression^25^. MCF-7-Y1 cells were cultivated in MEM Earle´s liquid medium with 2.2 g/L NaHCO_3_ (Merck Biochrom, Darmstadt) containing 10% FBS and 1% *L*-glutamine. All cells were cultured under sterile conditions in a humidified atmosphere containing 5% CO_2_ at 37 °C. Cells were routinely passaged twice a week.

#### Cellular accumulation assay

The cellular accumulation of Y_1_R radioligands by MCF-7-Y1 cells was measured: On the day prior to the experiment, 300,000 cells per well were seeded into 24-well plates (Greiner Bio-One). Attached cells were washed twice with cold PBS and 450 µL of assay medium (cell culture medium containing 0.1% BSA) were added to the cells for total binding. For non-specific binding, 400 µL of assay medium and 50 µL of medium containing 10 µM BIBP3226 were added. Finally, 50 µL of medium containing approx. 15 kBq of the respective radiotracer were added to each well, and cells were incubated at 37 °C for 10, 30, 60 or 90 min. After incubation, cells were washed with 500 µL of ice-cold PBS and lysed with 600 µL of warm 1 M NaOH. The lysed cells were transferred to counting tubes and analysed in the γ-counter (Wallac Wizard, Perkin Elmer, Waltham, MA, USA). Separate tubes containing 50 µL of medium with the radiotracer (approx. 15 kBq) were analysed as reference samples. Experiments were performed in sextuplicate (n = 6).

#### *In-vitro* autoradiography

MCF-7-Y1 tumour bearing mice were sacrificed by cervical dislocation under deep isoflurane anesthesia and tumours were excised and subsequently frozen in a hexane/dry ice bath (−70 °C). MCF-7-Y1 tumour slices (14 µm) were prepared on a cryostat microtome HM 500 O (Microm, Walldorf) and thaw-mounted on Histobond® adhesive glass slides (Marienfeld, Lauda-Königshofen).

For autoradiography studies slides were thawed and pre-incubated for 15 min in incubation buffer (50 mM TRIS HCl, pH 7.4, containing 120 mM NaCl, 5 mM MgCl_2_,) at room temperature. Thereafter, 800 µL of incubation buffer, containing 0.1 MBq of the respective radiotracer were pipetted on the slide and incubated at room temperature for 60 min. For displacement studies BIBP3226 (1 µM or 10 µM) was added to the incubation buffer before pipetting on the slides. Afterwards slides were washed by placing in ice cold incubation buffer (3 × 2 min) followed by short dipping in ice cold distilled water. Slides were carefully dried in a stream of warm air and finally placed on an autoradiography film (Fuji Imaging Plate BAS-IP SR 2025 E, Fujifilm, Düsseldorf) overnight prior to readout (25 µm resolution) on the autoradiograph (HD-CR-35 Bio, Raytest, Straubenhardt) and analysis with the software AIDA (Raytest). Additional sections were stained with hematoxylin and eosin (H&E) for comparison with the autoradiography images.

### *In-vivo* characterisation of radiotracers

All mouse experiments were approved by the local animal protection authorities (Government of Central Franconia, Germany, No. 55.2 2532-2-279) and performed at the FAU in accordance with the relevant E.U. guidelines and regulations.

#### Biodistribution

Biodistribution studies were conducted using female NMRI outbred mice (HsdWin:NMRI) purchased from Envigo (Horst, The Netherlands). Mice were kept in groups of four to five animals in individually ventilated cages in a twelve hours dark/light cycle with unlimited access to water and standard chow. At the age of eight to nine weeks six animals per radiotracer were injected with 2–4 MBq of the respective radiotracer under isoflurane anesthesia. Mice were sacrificed by cervical dislocation 30 or 90 min p.i. of the radiotracer. Blood as well as the organs/tissues lung, liver, kidneys, heart, spleen, brain, muscle, intestines, gall bladder and bones were harvested and analysed in the γ-counter. Samples were weighed and radioactivity in different tissues was calculated as percentage of the total injected dose per gram tissue (%ID/g).

#### Small animal PET

For the NPY Y_1_R xenograft model, female NMRI nude mice (HsdCpb:NMRI-Foxn1^nu^) were purchased from Envigo (Horst, The Netherlands) at the age of three weeks. Mice were kept in groups of four to five animals in individually ventilated cages in a twelve hours dark/light cycle with unlimited access to water and standard chow. At the age of nine to ten weeks one 17β-estradiol pellet per animal (0.72 mg per pellet, 3 mm diameter) with a 60-day release time (Innovative Research of America, Sarasota, FL, USA) was subcutaneously implanted on the back under isoflurane anesthesia. After three days, approximately 10^6^ MCF-7-Y1 tumour cells (in 50 µL PBS) were mixed with Matrigel (50 µL, BD Biosciences, Heidelberg) and then injected subcutaneously at the back. Tumour diameters and weight of the animals were recorded five times a week. Imaging studies were performed four weeks after inoculation of the cells.

Small animal PET scans were performed on an Inveon™ microPET scanner (Siemens Healthcare, Erlangen) under isoflurane anesthesia (3%). Tumour bearing mice (26–35 g, n = 2–3 for each radioligand) were intravenously injected into the tail vein with the respective radiotracer (1.5–4.2 MBq in about 100 µL of saline) under isoflurane anesthesia (3–4%). Static images were acquired for 15 min starting 45 min p.i. of the radiotracer. For displacement studies, the same mice were co-injected with the radiotracer (**[**^**18**^**F]12** and **[**^**18**^**F]13** only) and BIBP3226 (1 mg/kg). Images were corrected for decay and attenuation and MAP (iterative maximum a posteriori) images were reconstructed using the built-in software of the PET scanner. Evaluation of the MAP images was conducted using the software PMOD (version 3.6, PMOD Technologies LLC, Zürich). Regions of interests (ROIs) were drawn and radioactivity concentration within these regions was obtained from the mean values and calculated as percentage of the total injected dose per gram tissue (% ID/g).

#### Determination of radioactive metabolites of Y_1_R radioligands in mouse blood

Nude mice were anesthetised with isoflurane and injected with the respective radiotracer (3–25 MBq) into the tail vein. After 5 min, animals were sacrificed by cervical dislocation and approx. 100 µL blood were collected from the abdomen and transferred into Li-heparinised Microvettes® (100 LH, Sarstedt). The same volume of aqueous urea (0.8 g/mL) was added and the Microvette® was centrifuged (2000 × g, 5 min). The supernatant was transferred to a 1.5 mL reaction vial, mixed with the same volume of 10% aqueous TFA and centrifuged (20000 × g, 5 min). This step was repeated once. A sample of the resulting supernatant (100 µL) was analysed by radio-HPLC using two methods (Chromolith RP-18e, 100 × 4.6 mm, 4 mL/min, 0–1 min 25% CH_3_CN (0.1%TFA) in water (0.1%TFA), 1–6 min 25–60%, 6–7 min 60–100%, 7–8 min 100%; Kromasil 100 C8 5 µm, 250 × 4.6 mm, 1.5 mL/min, 1–25 min 20–53.3% CH_3_CN (0.1% TFA) in water (0.1% TFA), 25–27 min 53.3–80%, 27–28 min 80–100%, 28–32 min 100%).

## Supplementary information

Supplementary Information

## Data Availability

All data generated or analysed during this study are included in this published article (and its Supplementary Information files). Parts of this study have been reported in the PhD thesis ‘Selective neuropeptide and opioid receptor radioligands for imaging studies *in vivo* by positron emission tomography (PET)’ by Julian J. Ott^26^.

## References

[1] Holzer P, Reichmann F, Farzi A (2012). Neuropeptide Y, peptide YY and pancreatic polypeptide in the gut-brain axis. Neuropeptides.

[2] Blomqvist AG, Herzog H (1997). Y-receptor subtypes - how many more?. Trends Neurosci..

[3] Larhammar D, Wraith A, Berglund MM, Holmberg SKS, Lundell I (2001). Origins of the many NPY-family receptors in mammals. Peptides.

[4] Wraith A (2000). Evolution of the neuropeptide Y receptor family: gene and chromosome duplications deduced from the cloning and mapping of the five receptor subtype genes in pig. Genome Res..

[5] Lindner D, Stichel J, Beck-Sickinger AG (2008). Molecular recognition of the NPY hormone family by their receptors. Nutrition.

[6] Körner M, Waser B, Reubi JC (2003). Neuropeptide Y receptor expression in human primary ovarian neoplasms. Lab. Invest..

[7] Körner M, Waser B, Reubi JC (2004). High expression of neuropeptide Y receptors in tumors of the human adrenal gland and extra-adrenal paraganglia. Clin. Cancer. Res..

[8] Körner M, Waser B, Reubi JC (2005). Neuropeptide Y receptors in renal cell carcinomas and nephroblastomas. Int. J. Cancer.

[9] Reubi JC, Gugger M, Waser B, Schaer JC (2001). Y_1_-mediated effect of neuropeptide Y in cancer: breast carcinomas as targets. Cancer Res..

[10] Söll RM, Dinger MC, Lundell I, Larhammer D, Beck-Sickinger AG (2001). Novel analogues of neuropeptide Y with a preference for the Y_1_-receptor. Eur. J. Biochem..

[11] Khan IU (2010). Breast-cancer diagnosis by neuropeptide Y analogues: from synthesis to clinical application. Angew. Chem. Int. Ed..

[12] Hofmann S, Maschauer S, Kuwert T, Beck-Sickinger AG, Prante O (2015). Synthesis and *in vitro* and *in vivo* evaluation of an ^18^F-labeled neuropeptide Y analogue for imaging of breast cancer by PET. Mol. Pharm..

[13] Rudolf K (1994). The first highly potent and selective non-peptide neuropeptide Y Y_1_ receptor antagonist: BIBP3226. Eur. J. Pharmacol..

[14] Wieland HA, Engel W, Eberlein W, Rudolf K, Doods HN (1998). Subtype selectivity of the novel nonpeptide neuropeptide Y Y_1_ receptor antagonist BIBO 3304 and its effect on feeding in rodents. Br. J. Pharmacol..

[15] Hipskind PA (1997). Potent and selective 1,2,3-trisubstituted indole NPY Y_1_ antagonists. J. Med. Chem..

[16] Hostetler ED (2011). Synthesis, characterization, and monkey positron emission tomography (PET) studies of [^18^F]Y1-973, a PET tracer for the neuropeptide Y Y_1_ receptor. NeuroImage.

[17] Van Der Born, D. *et al*. Fluorine-18 labelled building blocks for PET tracer synthesis. **46**, 4709–4773 (2017). 10.1039/c6cs00492j 28608906

[18] Keller M (2017). Prototypic ^18^F-labeled argininamide-type neuropeptide Y Y_1_R antagonists as tracers for PET imaging of mammary carcinoma. ACS Med. Chem. Lett..

[19] Keller M (2008). Guanidine-acylguanidine bioisosteric approach in the design of radioligands: synthesis of a tritium-Labeled *N*^G^-propionylargininamide ([^3^H]-UR-MK114) as a highly potent and selective neuropeptide Y Y_1_ receptor antagonist. J. Med. Chem..

[20] Maschauer S, Haubner R, Kuwert T, Prante O (2014). ^18^F-Glyco-RGD peptides for PET imaging of integrin expression: efficient radiosynthesis by click chemistry and modulation of biodistribution by glycosylation. Mol. Pharm..

[21] Maschauer S (2015). Improved radiosynthesis and preliminary *in vivo* evaluation of a ^18^F-labeled glycopeptide-peptoid hybrid for PET imaging of neurotensin receptor 2. Bioorg. Med. Chem..

[22] Keller M (2015). *N*^ω^-Carbamoylation of the argininamide moiety: an avenue to insurmountable NPY Y_1_ receptor antagonists and a radiolabeled selective high-affinity molecular tool ([^3^H]UR-MK299) with extended residence time. J. Med. Chem..

[23] Kuhn KK (2016). High affinity agonists of the neuropeptide Y (NPY) Y_4_ receptor derived from the C-terminal pentapeptide of human pancreatic polypeptide (hPP): synthesis, stereochemical discrimination and radiolabeling. J. Med. Chem..

[24] Cheng Y, Prusoff WH (1973). Relationship between the inhibition constant (K1) and the concentration of inhibitor which causes 50 per cent inhibition (I50) of an enzymatic reaction. Biochem. Pharmacol..

[25] Keller M (2011). Red-fluorescent argininamide-type NPY Y_1_ receptor antagonists as pharmacological tools. Biorg. Med. Chem..

[26] Ott, J. J. *Selective neuropeptide and opioid receptor radioligands for imaging studies in vivo by positron emission tomography (PET)*, PhD thesis, Friedrich-Alexander-Universität Erlangen-Nürnberg (FAU), urn:nbn:de:bvb:29-opus4-99474 (2018).

