# Supplementary material for: 18F-labelled triazolyl-linked argininamides targeting the neuropeptide Y Y1R for PET imaging of mammary carcinoma
Source: Sci Rep. 2019 Sep 10;9:12990. doi: 10.1038/s41598-019-49399-0 (PMC6736837; doi:10.1038/s41598-019-49399-0)
Supplement: Supplementary file 1 — Supplementary Information [file 41598_2019_49399_MOESM1_ESM.pdf]

Supplementary Information

^{18}F-labelled triazolyl-linked argininamides targeting the neuropeptide Y Y_1R for PET imaging of mammary carcinoma

Simone Maschauer[1,‡], Julian J. Ott[1,‡], Günther Bernhardt[2], Torsten Kuwert[1], Max Keller[2], Olaf Prante[1,*]

[1]Department of Nuclear Medicine, Molecular Imaging and Radiochemistry, Friedrich-Alexander University (FAU), Schwabachanlage 6, 91054 Erlangen, Germany,

[2]Institute of Pharmacy, Faculty of Chemistry and Pharmacy, University of Regensburg, Universitätsstrasse 31, 93053 Regensburg, Germany

[‡] contributed equally

[*]Corresponding author: Prof. Dr. Olaf Prante, Molecular Imaging and Radiochemistry, Nuclear Medicine Clinic, Friedrich-Alexander University (FAU), Schwabachanlage 6, D-91054 Erlangen, Germany. Tel: +49-9131-8544440; Fax: +49-9131-8539288, E-mail: olaf.prante@uk-erlangen.de.

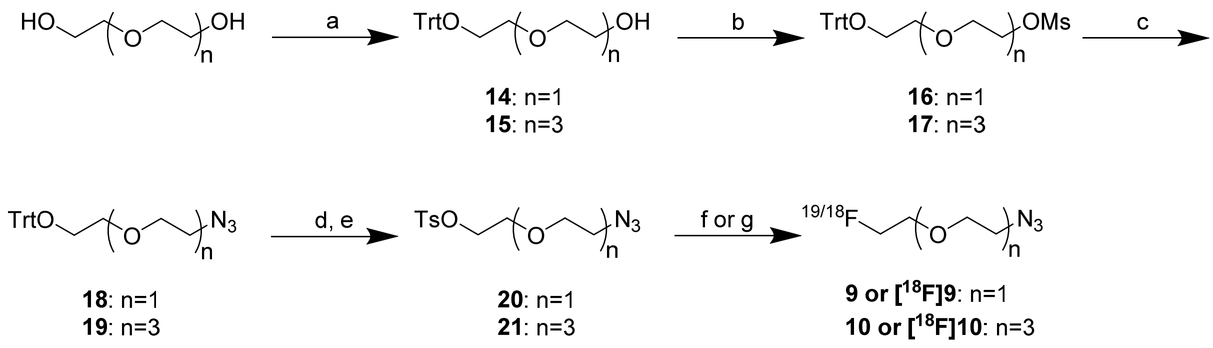

Scheme S1. Reaction scheme of the synthesis of labelling precursors **20** and **21** and their fluorine-bearing analogues **9** and **10**. Reagents: a) TrtCl, Et_3N, CH_2Cl_2, 85% (**14**), 83% (**15**); b) MsCl, Et_3N, CH_2Cl_2, quant. (**16**), 99% (**17**); c) NaN_3, CH_3CN, 83% (**18**), 67% (**19**); d) TsOH, CH_3OH; e) TsCl, Et_3N, CH_2Cl_2, 52% (**20**) 18% (**21**) f) TBAF, THF, 22% (**9**), 83% (**10**); g) Kryptofix® 2.2.2, K_2CO_3, KH_2PO_4, CH_3CN, 38% RAY for **[^{18}F]9**, 40% RAY for **[^{18}F]10**.

2-[2-(Triphenylmethoxy)ethoxy]ethan-1-ol (14)

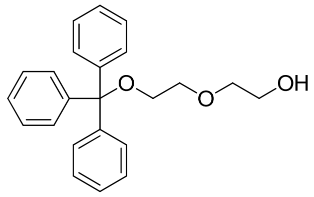

The synthesis was performed according to a protocol derived from Pilkington-Miksa et al.[1]. Diethylene glycol (63.7 g, 600 mmol, 20 eq.) was mixed with 200 mL dichloromethane in a round bottom flask above molecular sieve under nitrogen and triethylamine (8.4 mL, 60 mmol, 2 eq.) was added. Triphenylmethyl chloride (8.36 g, 30 mmol, 1 eq.) was dissolved in 60 mL dichloromethane, added dropwise during 90 min, and the mixture was stirred at room temperature overnight under nitrogen. The solvent was removed *in vacuo* to afford a slightly yellow oil which was dissolved in 300 mL dichloromethane. The solution was washed with saturated $NaHCO_3$ (200 mL), water (4× 250 mL) and brine (200 mL) and dried with Na_2SO_4. The solvent was removed to afford the crude product which was purified by flash column chromatography (eluent: dichloromethane → dichloromethane/methanol 99:1 v/v).

Yield: 8.9 g (85%) as a white crystalline solid.

TLC: ethyl acetate/hexane (1:1 v/v), $R_f = 0.56$

Dichloromethane/methanol (99:1 v/v), $R_f = 0.41\text{-}0.59$

staining with anisaldehyde reagent

^{1}H-NMR: (600 MHz, chloroform-d) δ 7.51-7.45 (m, 6H, AA’BB’), 7.34-7.29 (m, 6H, AA’BB’), 7.28-7.22 (m, 3H, AA’BB’C), 3.79-3.73 (m, 2H, CH_2), 3.71-3.68 (m, 2H, CH_2), 3.66-3.62 (m, 2H, CH_2), 3.29 (dd, J = 5.6, 4.5 Hz, 2H, CH_2), 2.11 (t, J = 6.1 Hz, 1H, OH).

2-[2-(Triphenylmethoxy)ethoxy]ethyl methanesulfonate (16)

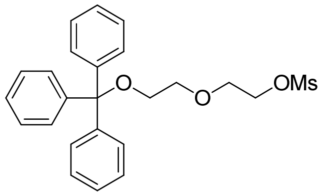

The synthesis was performed according to a protocol derived from Pilkington-Miksa et al.[1]. **14** (8.1 g, 23.2 mmol, 1 eq.) was dissolved in 100 mL dichloromethane in a round bottom flask above molecular sieve under nitrogen. The solution was cooled to 0 °C in an ice bath and triethylamine (9.6 mL, 69.6 mmol, 3 eq.) was added. Methanesulfonyl chloride (4.5 mL, 58 mmol, 2.5 eq.) was dissolved in 40 mL dichloromethane and added dropwise to the solution. The mixture was allowed to warm to room temperature and was stirred for 2.5 h. 50 mL saturated $NaHCO_3$ was added and the mixture was stirred for 10 min. Dichloromethane (200 mL) was added and the organic layer was washed with 100 mL brine. The combined aqueous layers were re-extracted with 150 mL dichloromethane. The organic layers were pooled and dried with Na_2SO_4. The solvent was removed *in vacuo* to afford the product as an orange oil which was used for the subsequent reactions without further purification.

Yield: 9.9 g (quant.) as an orange oil.

TLC: ethyl acetate/hexane (1:1 v/v), $R_f = 0.65$

staining with anisaldehyde reagent

^{1}H-NMR: (360 MHz, chloroform-d) δ 7.49-7.42 (m, 6H, AA’BB’), 7.35-7.27 (m, 6H, AA’BB’), 7.27-7.20 (m, 3H, AA’BB’C), 4.44-4.37 (m, 2H, O-CH_2-CH_2-O-Ms), 3.82-3.77 (m, 2H, O-CH_2-CH_2-O-Ms), 3.71-3.65 (m, 2H, O-CH_2-CH_2-O-C-$(Ph)_3$), 3.30-3.23 (m, 2H, O-CH_2-CH2-O-C-$(Ph)_3$), 3.01 (s, 3H, CH_3-Ms).

{[2-(2-Azidoethoxy)ethoxy]diphenylmethyl}benzene (18)

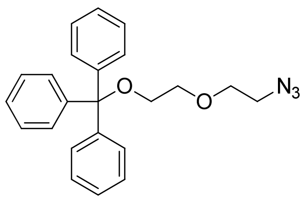

The synthesis was performed according to a protocol derived from Tahtaoui et al.[2]. **16** (10.7 g, 25.0 mmol, 1 eq.) was dissolved in 150 mL acetonitrile in a round bottom flask above molecular sieve. Sodium azide (2.4 g, 37.5 mmol, 1.5 eq.) was added and the mixture was refluxed for 48 h. The reaction was quenched with 200 mL water and the mixture was extracted with 150 mL dichloromethane. The aqueous layer was re-extracted with 100 mL dichloromethane. The combined organic layers were dried with $MgSO_4$ and the solvent was evaporated *in vacuo*. The resulting crude product was purified by flash column chromatography (eluent: ethyl acetate/hexane 1:1 v/v).

Yield: 7.8 g (83%) as a colorless oil.

TLC: ethyl acetate/hexane (1:1 v/v), $R_f = 0.90$

staining with anisaldehyde reagent

^{1}H-NMR: (360 MHz, chloroform-d) δ 7.55-7.41 (m, 6H, AA'BB'), 7.34-7.27 (m, 6H, AA'BB'), 7.27-7.21 (m, 3H, AA'BB'C), 3.70 (m, 4H, CH_2), 3.41 (m, 2H, CH_2-O-C-$(Ph)_3$ or O-CH_2-CH_2-N_3), 3.28 (m, 2H, CH_2-O-C-$(Ph)_3$ or O-CH_2-CH_2-N_3).

1-{[2-(2-Azidoethoxy)ethoxy]sulfonyl}-4-methylbenzene (20)

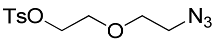

The synthesis was performed according to a protocol derived from Tahtaoui et al.[2]. **18** (7.2 g, 19.3 mmol, 1 eq.) was dissolved in 160 mL methanol, *p*-toluenesulfonic acid (332 mg, 1.93 mmol, 0.1 eq.) was added and the solution was refluxed for 4 h. Na_2CO_3 (4.1 g, 38.6 mmol, 2 eq.) was added and the mixture was stirred for another 5 min prior to removal of the solvent *in vacuo*. The residue was taken up in dichloromethane (100 mL) and filtered. The filtrate was cooled to 0 °C on an ice bath under nitrogen above molecular sieve and triethyl amine (5.4 mL, 38.6 mmol, 2 eq.) was added. *p*-Toluenesulfonyl chloride (9.8 g, 51.4 mmol, 2.7 eq.) was dissolved in 60 mL dichloromethane and was added dropwise. After 2 h the solution was allowed to warm to room temperature and was stirred for 72 h. The resulting precipitate was filtered off and the filtrate was concentrated *in vacuo*. The crude product was purified by flash column chromatography (eluent: ethyl acetate/hexane 1:2 v/v).

Yield: 2.9 g (52%) as a yellow oil.

TLC: ethyl acetate/hexane (1:2 v/v), $R_f = 0.45$

ethyl acetate/hexane (2:1 v/v), $R_f = 0.77$

staining with anisaldehyde reagent

^{1}H-NMR: (360 MHz, chloroform-d) δ 7.83-7.78 (m, 2H, AA'BB'), 7.39-7.32 (m, 2H, AA'BB'), 4.22-4.14 (m, 2H, CH_2), 3.76-3.65 (m, 2H, CH_2), 3.60 (m, 2H, CH_2), 3.32 (t, J = 5.0 Hz, CH_2), 2.45 (s, 3H, CH_3-Ts).

1-Azido-2-(2-fluoroethoxy)ethane (9)

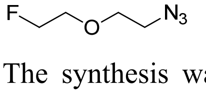

The synthesis was performed according to a protocol derived from Breyholz et al.[3]. **20** (860 mg, 3.0 mmol, 1 eq.) was dissolved in a 1 M solution of tetrabutylammonium fluoride in tetrahydrofuran (4.5 mL, 4.5 mmol, 1.5 eq.) and refluxed for 2 h. The mixture was allowed to cool to room temperature and stirred overnight. The reaction was quenched with 21 mL water and the mixture was extracted with 50 mL dichloromethane. The organic layer was dried with Na_2SO_4 and the solvent was removed *in vacuo*.

Yield: 87 mg (22%) as a slightly yellow oil

^{1}H-NMR: (360 MHz, chloroform-d) δ 4.68-4.47 (m, 2H, F-CH_2-CH_2), 3.83-3.36 (m, 6H, CH_2).

1,1,1-Triphenyl-2,5,8,11-tetraoxatridecan-13-ol (15)

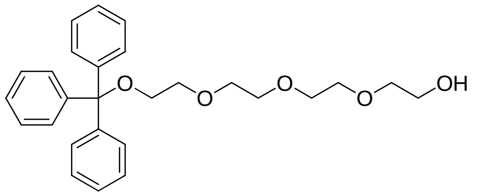

The synthesis was performed according to the protocol by Pilkington-Miksa et al.[1]. Tetraethylene glycol (77.7 g, 400 mmol, 20 eq.) was mixed with 250 mL dichloromethane in a round bottom flask above molecular sieve under nitrogen and triethylamine (5.6 mL, 40 mmol, 2 eq.) was added. Triphenylmethyl chloride (5.58 g, 20 mmol, 1 eq.) was dissolved in 60 mL dichloromethane, added dropwise during 90 min and the mixture was stirred at room temperature overnight under nitrogen. The solvent was removed *in vacuo* to afford a slightly yellow oil which was dissolved in 270 mL dichloromethane. The solution was washed with saturated $NaHCO_3$ (135 mL), water (4× 170 mL) and brine (170 mL) and dried with Na_2SO_4. The solvent was removed to afford the crude product which was purified by flash column chromatography (eluent: ethyl acetate/hexane 9:1 v/v).

Yield: 7.2 g (83%) as a slightly yellow oil.

TLC: ethyl acetate/hexane (9:1 v/v), R_f = 0.50
dichloromethane/methanol (95:5 v/v), R_f = 0.65
staining with anisaldehyde reagent

^{1}H-NMR: (360 MHz, chloroform-d) δ 7.52-7.18 (m, 15H, Ph), 3.75-3.63 (m, 12H, CH_2), 3.63-3.56 (m, 2H, CH_2-OH), 3.25 (t, J = 5.3 Hz, 2H, CH_2-O-C-$(Ph)_3$), 2.50-2.30 (m, 1H, OH).

1,1,1-Triphenyl-2,5,8,11-tetraoxatridecan-13-yl methanesulfonate (17)

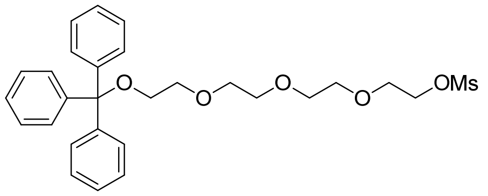

The synthesis was performed according to a protocol derived from Pilkington-Miksa et al.[1]. **15** (7.2 g, 16.5 mmol, 1 eq.) was dissolved in 70 mL dichloromethane in a round bottom flask above molecular sieve under nitrogen. The solution was cooled to 0 °C in an ice bath and triethylamine (8.3 mL, 60 mmol, 3.6 eq.) was added. Methanesulfonyl chloride (3.9 mL, 50 mmol, 3 eq.) was dissolved in 30 mL dichloromethane and added dropwise to the solution. The mixture was allowed to warm to room temperature and was stirred for 2.5 h. Saturated $NaHCO_3$ (50 mL) was added and the mixture was

stirred for 10 min. 200 mL dichloromethane was added and the organic layer was washed with 100 mL brine. The combined aqueous layers were re-extracted with 150 mL dichloromethane. The organic layers were pooled and dried with Na_2SO_4.. The solvent was removed *in vacuo* to afford the product as an orange oil which was used for the subsequent reactions without further purification.

Yield: 8.4 g (99%) as an orange oil.

TLC: ethyl acetate/hexane (1:1 v/v), $R_f = 0.30$

staining with anisaldehyde reagent

^{1}H-NMR: (600 MHz, chloroform-d) δ 7.50-7.45 (m, 6H, AA'BB'), 7.33-7.28 (m, 6H, AA'BB'), 7.28-7.22 (m, 3H, AA'BB'C), 4.37-4.32 (m, 2H, O-CH_2-CH_2-O-Ms), 3.80-3.73 (m, 2H, O-CH_2-CH_2-O-Ms), 3.72-3.63 (m, 10H, CH_2), 3.25 (t, J = 5.3 Hz, 2H, CH_2-O-C-$(Ph)_3$), 3.00 (s, 3H, CH_3-Ms).

13-Azido-1,1,1-triphenyl-2,5,8,11-tetraoxatridecane (19)

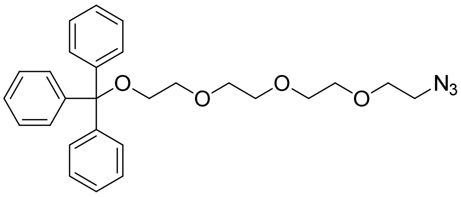

The synthesis was performed according to a protocol derived from Tahtaoui et al.[2]. **17** (8.4 g, 16.3 mmol, 1 eq.) was dissolved in 150 mL acetonitrile in a round bottom flask above molecular sieve. Sodium azide (1.43 g, 22.0 mmol, 1.35 eq.) was added and the mixture was refluxed for 40 h. The reaction was quenched with 200 mL water and the mixture was extracted with 150 mL dichloromethane. The aqueous layer was re-extracted with 100 mL dichloromethane. The combined organic layers were dried with $MgSO_4$ and the solvent was evaporated *in vacuo*. The resulting crude product was purified by column chromatography (eluent: ethyl acetate/hexane 1:1 v/v).

yield: 5.0 g (67%) as an orange oil.

TLC: ethyl acetate/hexane (1:1 v/v), $R_f = 0.81$

staining with anisaldehyde reagent

^{1}H-NMR: (360 MHz, chloroform-d) δ 7.50-7.44 (m, 6H, AA'BB'), 7.34-7.26 (m, 6H, AA'BB'), 7.25-7.20 (m, 3H, AA'BB'C), 3.76-3.56 (m, 12H, CH_2), 3.35 (t, J = 5.2 Hz, 2H, CH_2-O-C-$(Ph)_3$ or O-CH_2-CH_2-N_3), 3.25 (t, J = 5.4 Hz, 2H, CH_2-O-C-$(Ph)_3$ or O-CH_2-CH_2-N_3).

1-[(2-{2-[2-(2-Azidoethoxy)ethoxy]ethoxy}ethoxy)sulfonyl]-4-methylbenzene (21)

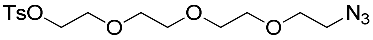

The synthesis was performed according to a protocol derived from Tahtaoui et al.[2]. **19** (5.0 g, 10.8 mmol, 1 eq.) was dissolved in 100 mL methanol, *p*-toluenesulfonic acid (190 mg, 1.1 mmol, 0.1 eq.) was added and the solution was refluxed for 4 h. Na_2CO_3 (2.3 g, 21.7 mmol, 2 eq.) was added

and the mixture was stirred for another 5 min prior to removal of the solvent *in vacuo*. The residue was taken up in dichloromethane (100 mL) and filtered. The filtrate was cooled to 0 °C on an ice bath under nitrogen above molecular sieve. Triethyl amine (3 mL, 21.7 mmol, 2 eq.) and *p*-toluenesulfonyl chloride (4.1 g, 21.7 mmol, 2 eq.) were added and the mixture was stirred at 0 °C. After 2 h the solution was allowed to warm to room temperature and was stirred overnight. After 4 and again after 10 d of stirring *p*-toluenesulfonyl chloride (2.0 g, 10.5 mmol, 1 eq.) was added. After stirring for another 24 h the resulting precipitate was filtered off and the filtrate was concentrated *in vacuo*. The crude product was purified by flash column chromatography (eluent: ethyl acetate/hexane 2:1 v/v).
Yield: 720 mg (18%) as a yellow oil.

TLC: ethyl acetate/hexane (2:1 v/v), $R_f = 0.65$
staining with anisaldehyde reagent

^{1}H-NMR: (600 MHz, chloroform-d) δ 7.83-7.78 (m, 2H, AA'BB'), 7.37-7.33 (m, 2H. AA'BB'), 3.72-3.58 (m, 14H, CH_2), 3.39 (t, J = 5.1 Hz, 2H, CH_2), 2.46 (s, 3H, CH_3-Ts).

1-Azido-2-{2-[2-(2-fluoroethoxy)ethoxy]ethoxy}ethane (10)

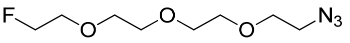

The synthesis was performed according to a protocol derived from Breyholz et al.[3]. **21** (200 mg, 0.54 mmol, 1 eq.) was dissolved in a 1 M solution of tetrabutylammonium fluoride in tetrahydrofuran (0.8 mL, 0.8 mmol, 1.5 eq.) and refluxed for 2 h. The mixture was allowed to cool to room temperature and stirred overnight. The reaction was quenched with 4 mL water and the mixture was extracted twice with 10 mL dichloromethane each. The organic layer was washed with water (2× 8 mL) and dried with Na_2SO_4. The solvent was removed *in vacuo* and the resulting crude product was purified by column chromatography (eluent: ethyl acetate/hexane 1:2 v/v).
Yield: 100 mg (83%) as a slightly yellow oil.

TLC: ethyl acetate/hexane (1:2 v/v), $R_f = 0.61$
staining of plate with anisaldehyde reagent lead to colorless product spot

^{1}H-NMR: (600 MHz, chloroform-d) δ 4.63-4.51 (m, 2H, F-CH_2-CH_2), 3.80-3.72 (m, 2H, F-CH_2-CH_2), 3.72-3.66 (m, 12H, CH_2).

tert-Butyl N-[(1Z)-(methylsulfanyl)({[(prop-2-yn-1-yl)carbamoyl]imino})methyl]carbamate (5)

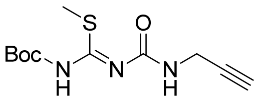

A 100 mL two-necked round bottom flask equipped with a pressure equalizing addition funnel was heated, evacuated and purged with argon prior to the reaction. A solution of propargyl amine (219 mg, 4 mmol, 2 eq) and *N*,*N*-diisopropylethyl amine (1440 mg, 11.1 mmol, 5.6 eq.) in 30 mL anhydrous dichloromethane were added dropwise to a stirred solution of triphosgene (591 mg, 2 mmol, 1 eq.) in anhydrous dichloromethane (15 mL) within 30 min. *t*-Butyl N-[(methylsulfanyl)methan-imidoyl]carbamate (759 mg, 4 mmol, 2 eq.) was added and the mixture was stirred at room temperature for 2.5 h. The solvent was evaporated and the crude product was purified by column chromatography (eluent: dichloromethane/ethyl acetate 50:1 v/v).

yield: 801 mg as a highly viscous yellow oil (74%)

TLC: ethyl acetate/hexane (1:1 v/v), $R_f = 0.82$

dichloromethane/ethyl acetate (50:1 v/v), $R_f = 0.83$

^{1}H-NMR: (360 MHz, DMSO-d_6) δ 12.18 (s, 1H, CO-NH-C), 8.15 (t, J = 5.9 Hz, 1H, CO-NH-CH_2), 3.81 (dd, J = 5.9, 2.5 Hz, 2H, CH_2), 3.07 (t, J = 2.5 Hz, 1H, C-CH), 2.28 (s, 3H, CH_3), 1.44 (s, 9H, *t*Bu).

Table S1: Structures, *in-vitro* Y_1 receptor affinities and selectivities of potential Y_1R PET radioligands. For Y_2R, Y_4R and Y_5R, K_i values are given in the first row. In the second row, the calculated selectivities for Y_1R over Y_2R, Y_4R and Y_5R are given as a multiple of the K_i value for Y_1R.

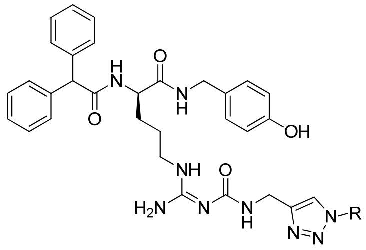

	compound structure		hY_1R	hY_2R	hY_4R	hY_5R
			$K_i \pm SEM^a$	K_i^b	K_i^c	K_i^b
				selectivity	selectivity	selectivity
**11**	R =	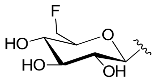	208 ± 24	> 10000<br>> 48 ×	> 3000<br>> 14 ×	> 15000<br>> 72 ×
**12**	R =	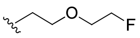	2.8 ± 0.7	> 10000<br>> 3610 ×	> 10000<br>> 3610 ×	> 15000<br>> 5415 ×
**13**	R =	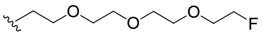	29.0 ± 3.6	> 5000<br>> 173 ×	> 10000<br>> 345 ×	> 10000<br>> 345 ×

[a]Determined by competition binding on SK-N-MC neuroblastoma cells using the radioligand [^{3}H]UR-MK299[4]. Mean K_i values ± SEM are the results of three individual experiments each performed in triplicate.
[b]Determined by competition binding at CHO-hY_2-G_{qi5}-mtAEQ cells and HEC-1b hY_5R cells using the radioligand [^{3}H]propionyl-pNPY. Two independent experiments were performed in triplicate.
[c]Determined by competition binding on CHO-hY_4R-G_{qi5}-mtAEQ cells using the radioligand [^{3}H]UR-KK200. Two independent experiments were performed in triplicate.
SEM = standard error of the mean.

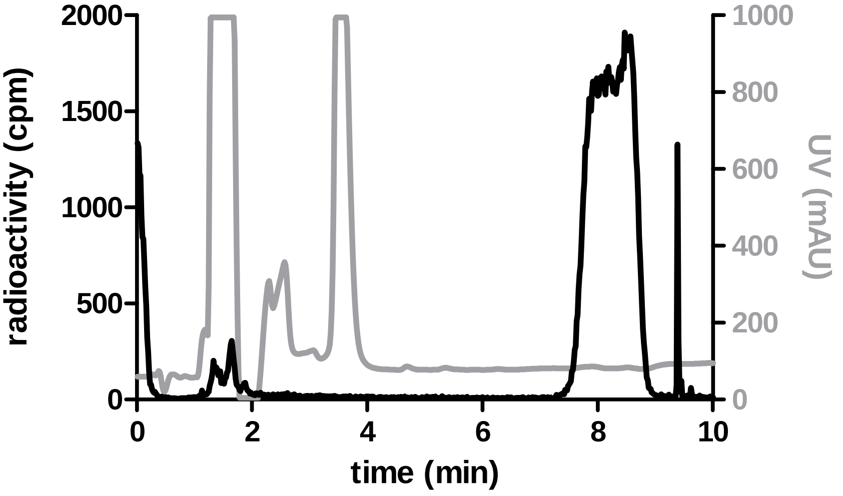

Figure S1: Semipreparative HPLC chromatogram of the isolation of **[^{18}F]9** (t_R = 8.1 min). Kromasil 100 C8 5 µm, 125×8.0 mm, 4 mL/min, 10-50% MeCN (0.1% TFA) in water (0.1% TFA) in 25 min. Figure adapted from corresponding PhD thesis[5].

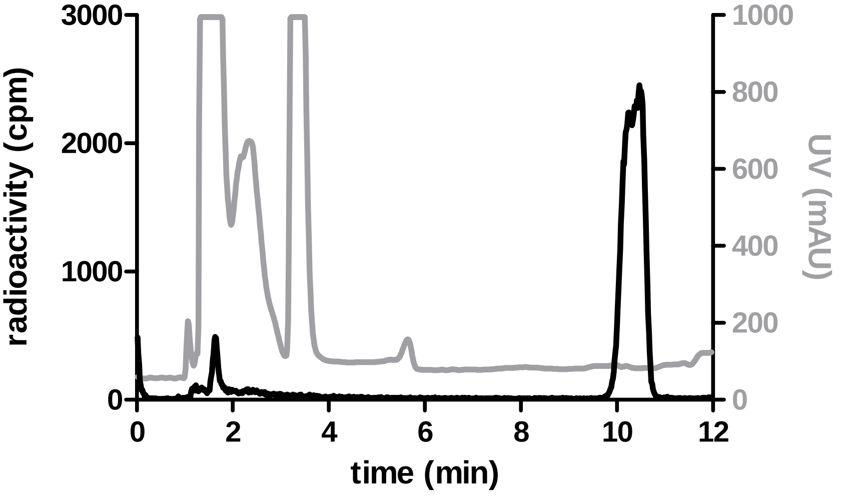

Figure S2: Semipreparative HPLC chromatogram of the isolation of **[^{18}F]10** (t_R = 10.3 min). Kromasil 100 C8 5 µm, 125×8.0 mm, 4 mL/min, 10-50% MeCN (0.1% TFA) in water (0.1% TFA) in 25 min. Figure adapted from corresponding PhD thesis[5].

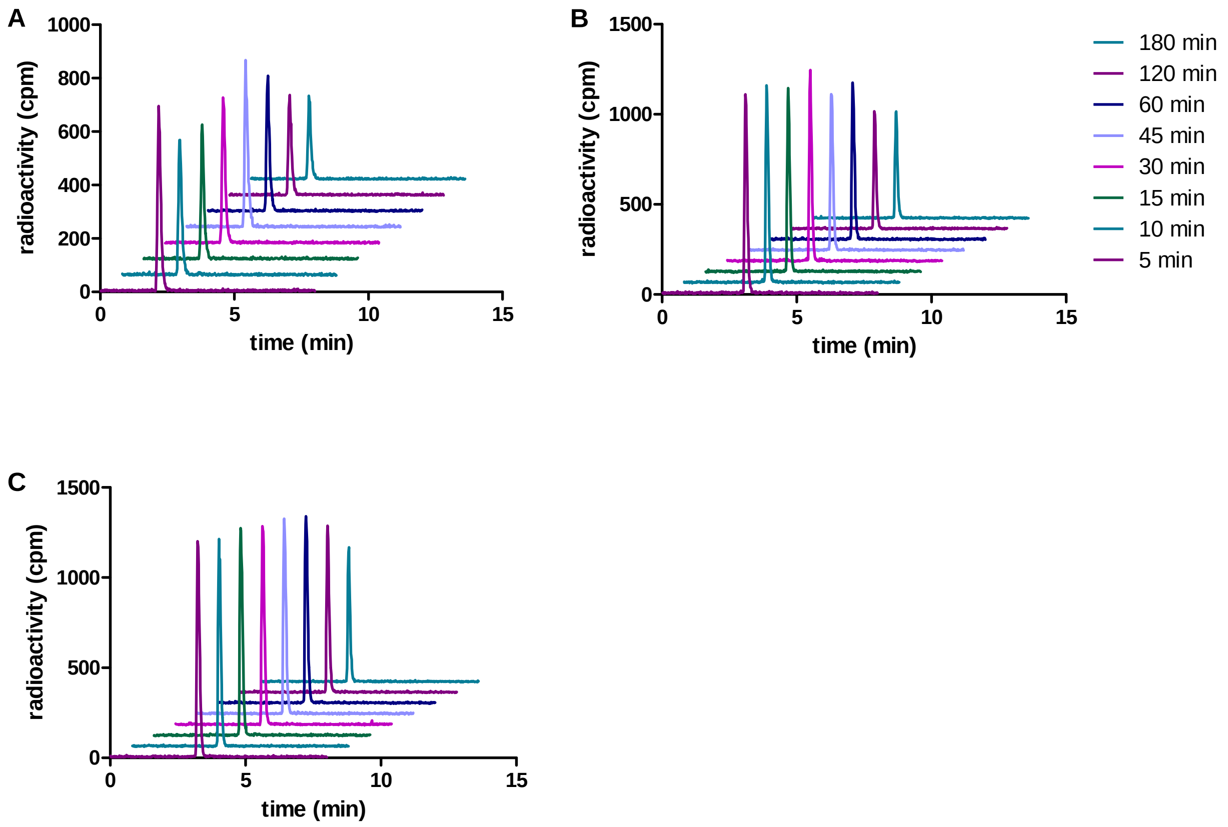

Figure S3: Stability of A) **[^{18}F]11** (t_R = 2.17 min), B) **[^{18}F]12** (t_R = 2.96 min) and C) **[^{18}F]13** (t_R = 3.21 min) *in vitro* in human serum, incubated at 37°C. Chromolith RP-18e, 100×4.6 mm, 4 mL/min, 0-1 min 25% CH_3CN (0.1% TFA) in water (0.1% TFA), 1-6 min 25-60%, 6-7 min 60-100%, 7-8 min 100%. Figure adapted from corresponding PhD thesis[5].

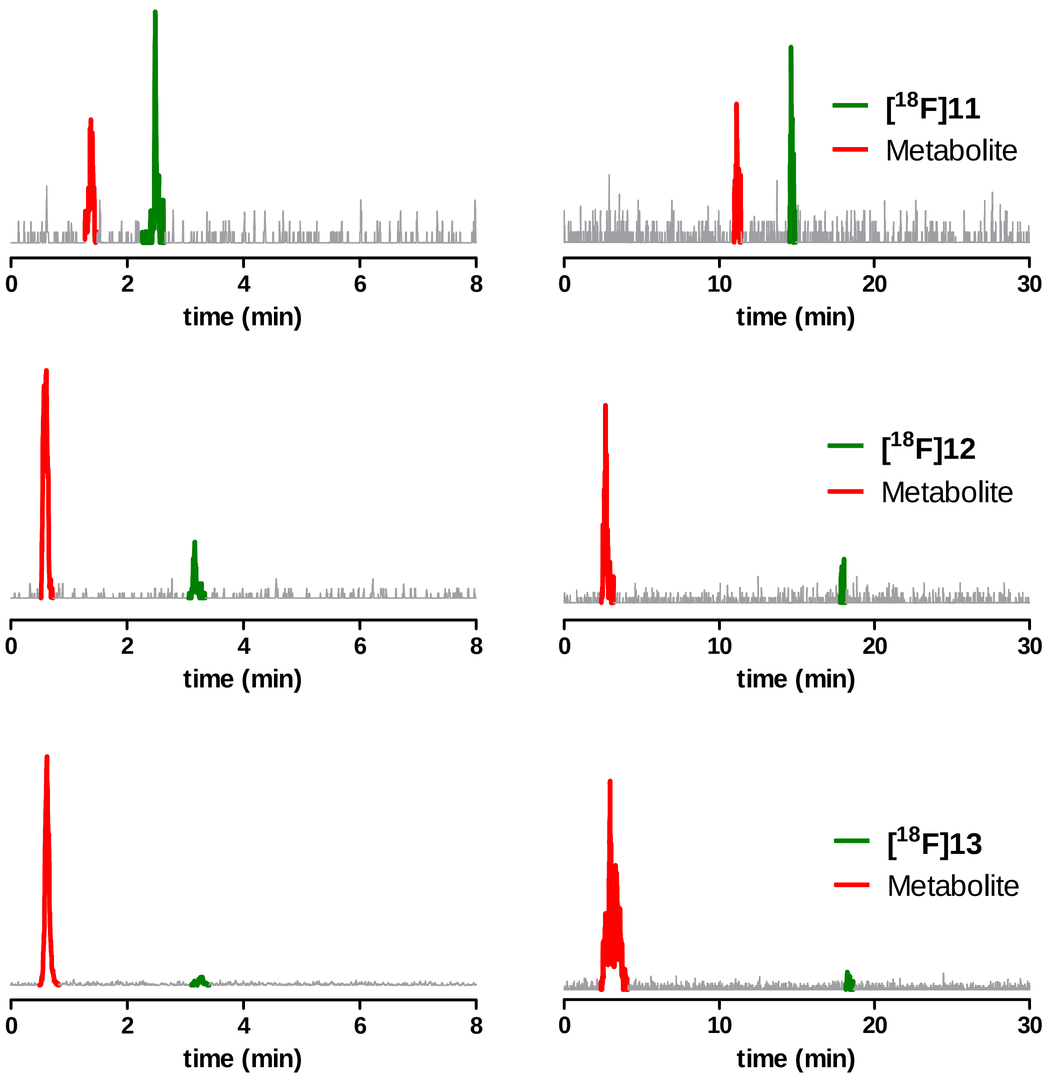

Figure S4: *Ex-vivo* stabilities determined by radio-HPLC from mouse blood samples taken 5 min after tracer injection. HPLC conditions: left side: Chromolith RP-18e, 100×4.6 mm, 4 mL/min, 0-1 min 25% CH_3CN (0.1% TFA) in water (0.1% TFA), 1-6 min 25-60%, 6-7 min 60-100%, 7-8 min 100%; right side: Kromasil 100 C8 5 µm, 250×4.6 mm, 1.5 mL/min, 1-25 min 20-53.3% CH_3CN (0.1% TFA) in water (0.1% TFA), 25-27 min 53.3-80%, 27-28 min 80-100%, 28-32 min 100%.

References:

1 Pilkington-Miksa, M. A. *et al.* Synthesis of bifunctional integrin-binding peptides containing PEG spacers of defined length for non-viral gene delivery. *Eur. J. Org. Chem.*, 2900-2914, doi:DOI 10.1002/ejoc.200701188 (2008).

2 Tahtaoui, C. *et al.* Fluorescent pirenzepine derivatives as potential bitopic ligands of the human M1 muscarinic receptor. *J. Med. Chem.* **47**, 4300-4315, doi:10.1021/jm040800a (2004).

3 Breyholz, H. J. *et al.* Radiofluorinated pyrimidine-2,4,6-triones as molecular probes for noninvasive MMP-targeted imaging. *ChemMedChem* **5**, 777-789, doi:10.1002/cmdc.201000013 (2010).

4 Keller, M. *et al.* N^{ω}-Carbamoylation of the argininamide moiety: an avenue to insurmountable NPY Y_1 receptor antagonists and a radiolabeled selective high-affinity molecular tool ([^{3}H]UR-MK299) with extended residence time. *J. Med. Chem.* **58**, 8834-8849, doi:10.1021/acs.jmedchem.5b00925 (2015).

5 Ott, J. J. *Selective neuropeptide and opioid receptor radioligands for imaging studies in vivo by positron emission tomography (PET)*, PhD thesis, Friedrich-Alexander-Universität Erlangen-Nürnberg (FAU), urn:nbn:de:bvb:29-opus4-99474, (2018).
